# Lattice oxygen-mediated ABO_3_-type NdFeO_3_ perovskite fabricated over multi-walled CNTs for the O_2_ evolution reaction and photo-induced degradation of Victoria blue B

**DOI:** 10.1039/d6ra05821c

**Published:** 2026-09-29

**Authors:** Rabbia Shoukat, Sadam Hussain, Sadia Ata, Hanaa A. Galeb, Aliya Kouser, Ayesha Sadiqa, Adnan Ashraf, Awais Ahmad

**Affiliations:** a School of Chemistry, University of the Punjab Lahore 54000 Pakistan sadia.chem@pu.edu.pk; b School of Natural Sciences (SNS), Department of Chemistry, NUST H12 Islamabad Pakistan; c Department of Chemistry, Faculty of Science and Arts, King Abdulaziz University Rabigh Saudi Arabia; d Institute of Chemistry, Khwaja Fareed University of Engineering and Information Technology Rahim Yar Khan Pakistan; e Department of Chemistry, The University of Lahore Lahore Pakistan awaisahmed@gcuf.edu.pk; f Faculty of Engineering and Technology, Shinawatra University Sam Khok Thailand

## Abstract

Hydrogen is generally considered a future sustainable energy carrier, and its electrochemical synthesis is based on the hydrogen evolution reaction (HER) and oxygen evolution reaction (OER). Nonetheless, a high energy barrier and slow reaction kinetics plague the OER, posing huge obstacles to efficient hydrogen production. In order to address the shortcoming, designing efficient electrocatalysts is essential to enhancing the efficiency and scalability of the OER. On the other hand, the emergence of life-threatening pollutants has pushed researchers towards designing photocatalysts for photo-induced degradation. Therefore, a perovskite (PSK) ABO_3_-type NdFeO_3_ was fabricated over multi-walled carbon nanotubes (MWCNTs) to target dual applications of water splitting and photodegradation. Techniques such as XRD, FTIR spectroscopy, SEM, TEM, EDS, PL/UV-vis spectroscopy, TGA, BET, and Raman spectroscopy were used to characterize the nanocomposite. The prepared NdFeO_3_@MWCNTs showed a direct band gap energy (*E*_g_) of 2.5 eV and an indirect *E*_g_ of 1.9 eV as determined through UV-visible spectroscopy. Furthermore, numerous parameters, including catalyst dosage, pollutant concentration, oxidizing agent, and pH, were comprehensively examined for the degradation of Victoria blue B dye. The percentage degradation was found to be optimized for the 250 ppm pollutant solution with 97% removal efficiency within the initial 5 minutes. Furthermore, the electrochemical studies of NdFeO_3_@MWCNTs showed its potential as an electrocatalyst with an overpotential (*η*) of 250 mV at a current density of 10 mA cm^−2^, along with a low Tafel slope of 67 mV dec^−1^. Moreover, the material showed an electrochemical surface area (ECSA) of 100 cm^2^ and a decreased charge transfer resistance (*R*_ct_) of 1.5 Ω, with the low *R*_ct_ being ascribed to the faster charge transfer rate. Hence, the low-cost NdFeO_3_@MWCNTs nanocomposite, compared to noble metal electrodes, is not only a promising photocatalyst but also an electrocatalyst for future applications in H_2_ generation.

## Abstract 2

Perovskite (PSK) ABO_3_-type NdFeO_3_ fabricated over multi-walled carbon nanotubes (MWCNTs) is being synthesized to target twin applications of water-splitting and photodegradation.

## Graphical abstract

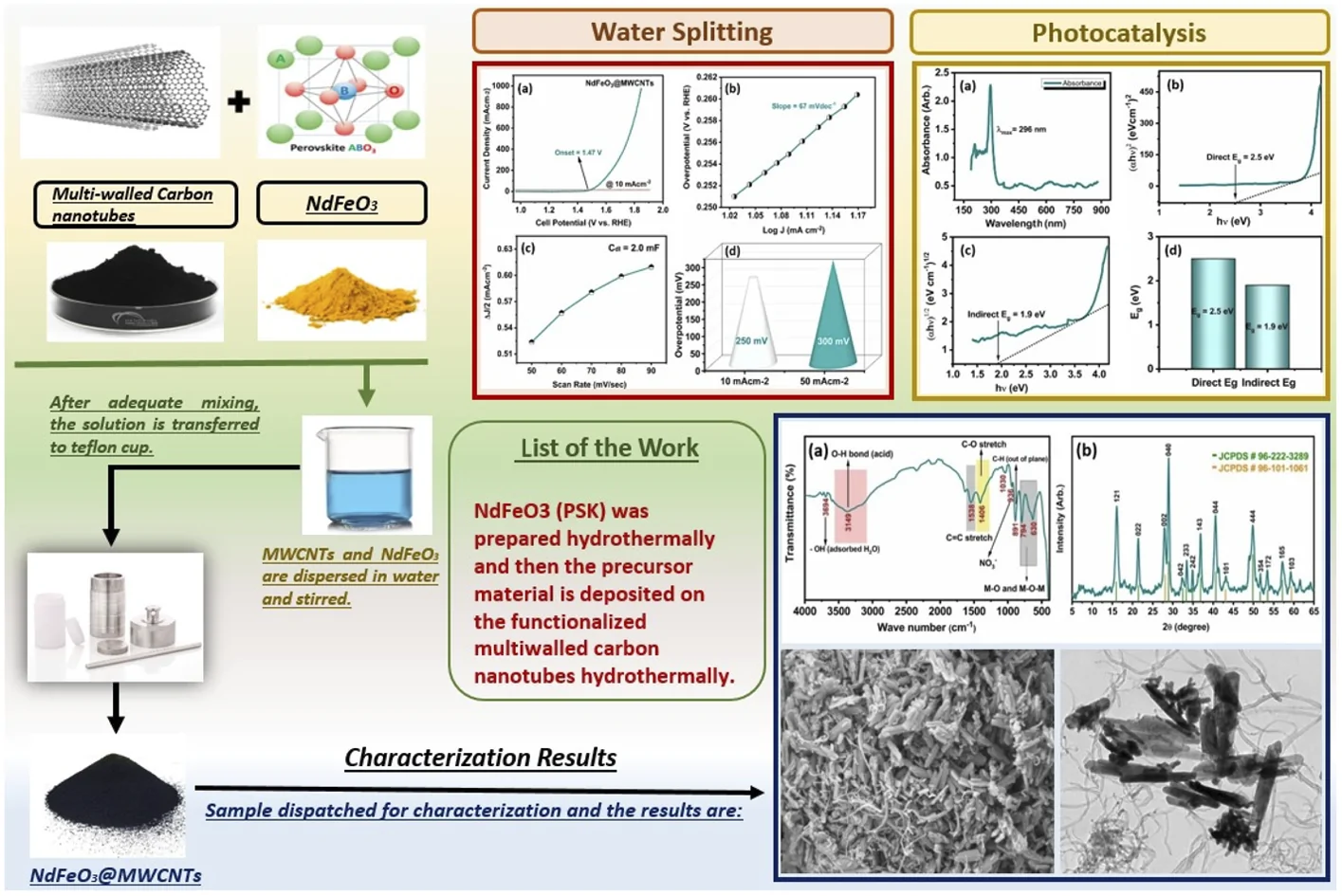

## 1. Introduction

Water, labelled as the universal solvent,^1^ is currently contaminated with life-threatening pollutants, making it unsuitable for consumption.^2^ These pollutants encompass not only industrial adulterants^3^ but also pharmaceutical toxins.^4^ Moreover, these pollutants are harmful to the life thriving on land and are equally detrimental to aquatic life as well.^5^ Therefore, there is a need to plan strategies that can eliminate these contaminants. For this, ultra-filtration,^6^ photocatalysis,^7^ biological methods,^8^ reverse osmosis,^9^ electrochemical sensing,^10^ ion exchange,^11^ and other methodologies are being developed. However, all these approaches come with some drawbacks. The ultra-filtration process suffers from membrane fouling and material accumulation on the membrane surface.^12^ Biological methods involve microorganisms in degradation processes and face the challenge of excessive microorganism requirements.^13^ A high rate of solute rejection in reverse osmosis is also a drawback.^14^ Ion exchange makes it a prerequisite that the components to be degraded must be charged under neutral conditions.^15^ Because of the shortcomings of conventional approaches, advanced oxidation processes (AOPs)^16^ are leading the course of action. AOPs can occur through diverse routes; among them, photo-Fenton is a much-praised approach owing to its low energy demand. In this process, H_2_O_2_ and Fe^3+^ ions are used. Fe^2+^ is first generated upon the reduction of Fe^3+^ under light irradiation, and then, ˙OH radicals, which are strong oxidizing agents, are produced when Fe^2+^ reacts with H_2_O_2_.^17^

1 Fe^3+^ + *hν*(sunlight) → Fe^2+^

2 Fe^2+^ + H_2_O_2_ → ˙OH + Fe^3+^ + H_2_O

Among various semiconductor photocatalysts, ABO_3_-type perovskite oxides have attracted considerable interest because of their structural versatility and excellent photocatalytic activity. Their intrinsic ability to regulate oxygen vacancies, band structures, and charge-transfer pathways provides opportunities for designing high-performance photocatalysts.^18^ In this regard, NdFeO_3_@MWCNTs were fabricated for evaluation as an electrocatalyst. Apart from fabrication, doping and compositing with layered structures are also ways to carry out defect creation, which enhances the overall material performance. Substitutional doping modifies the electronic structure based on the site that the dopant occupies, thereby enhancing efficiency.^19^ Recent studies have demonstrated that doping-induced defect engineering can regulate surface properties and introduce active sites, promoting reactive species generation and enhancing photocatalytic degradation efficiency.^20^ Furthermore, incorporation of dopants into semiconductor lattices has been reported to improve photocatalytic performance by facilitating interfacial charge transfer and suppressing electron–hole recombination pathways.^21^

Besides doping, constructing composite photocatalysts with layered materials has emerged as another promising approach because the formation of heterointerfaces can accelerate charge migration and reduce the recombination losses of photogenerated carriers.^22^ Layered composite architectures provide efficient pathways for electron transfer and improve photocatalytic stability by creating synergistic interactions between different components, resulting in enhanced degradation performance under irradiation.^23^

Among various perovskite oxides, NdFeO_3_ has attracted considerable attention due to its stable ABO_3_ framework, narrow band gap, and favorable electronic configuration arising from the interaction between the rare-earth Nd ions and the transition-metal Fe ions. The presence of Fe-based active centers provides potential redox activity, making NdFeO_3_ suitable for both photocatalytic and electrocatalytic applications. However, its practical application is limited by charge-carrier recombination and its relatively poor electrical conductivity. Therefore, coupling NdFeO_3_ with conductive carbon-based materials such as MWCNTs can provide efficient electron-transport pathways and enhance its overall catalytic performance.

However, our research focuses on designing MWCNT-doped NdFeO_3_ as a catalyst for Victoria blue B dye degradation, and its activity in both UV and visible regions^24^ supports its applicability as a photocatalyst. Neodymium, a potent lanthanide when modified into ferrite, enhances the compound's performance.^25^ However, it has a drawback, which is the agglomeration of NdFe_2_O_4_ due to its size.^24^ This needs to be tackled. Hence, a dopant is introduced to address the aggregation of the precursor material. MWCNTs have excellent electronic properties and outstanding mechanical tensile strength and are robust for the intended application.^26,27^ The degradation efficiency of the designed catalyst is enhanced using an activator, which is optimized as a parameter. For this purpose, H_2_O_2_ ^28^ proves to be an unmatched booster of activity. Hydroxyl radicals generated from H_2_O_2_ serve as an oxidizing agent.^29^ Ilanchezhiyan *et al.* synthesized a neodymium oxide perovskite nanomaterial for photo-induced electrochemical H_2_O splitting.^30^ Jasrotia *et al.* performed electrocatalytic hydrogen production by employing La/Nd-substituted cobalt–nickel nanomaterials.^31^ Adewuyi *et al.* synthesized NdFe_2_O_4_ over graphitic carbonitride (NdFe_2_O_4_@g-C_3_N_4_) for the photodegradation of ciprofloxacin (CIP) and ampicillin (AMP).^24^ In addition, Abbad *et al.* hydrothermally synthesized copper and gadolinium co-doped NdFe_2_O_4_ for crystal violet (CV) dye degradation and achieved 91.8% efficacy.^32^ Prabagar *et al.* hydrothermally synthesized NdFeO_3_ perovskite for methylene blue and lomefloxacin photocatalysis.^33^ Phan *et al.* prepared copper-substituted NdFeO_3_ by employing a hydrothermal technique and investigated the visible-light-assisted Fenton degradation process of phenolic compounds.^25^ Bhat *et al.* synthesized a copper-doped perovskite oxide (CaTiO_3_) for methylene blue degradation, and the results were exceptional due to the low band gap and decreased charge-carrier recombination.^34^ Uma *et al.* carried out the photodegradation of methyl violet dye using copper-doped SrTiO_3_ with above 90% degradation efficiency.^35^ The above-mentioned studies demonstrate that ample work has been carried out on NdFe_2_O_4_ as a photocatalyst for pollutant degradation. However, to the best of our knowledge, the designing of NdFe_2_O_4_@MWCNT as photocatalysts has not been reported to date. Hence, the effectiveness of the novel NdFe_2_O_4_@MWCNT nanocomposite for the photo-oxidation of an organic dye, along with H_2_O splitting, is clearly substantiated in the current study.

Synthesis is carried out hydrothermally through a facile route, which involves the preparation of NdFeO_3_. The functionalization of MWCNTs is a potent step. Functionalization promotes efficient dispersion and boosts electrical properties of the tubes.^36^

In the present study, a perovskite material was designed using a facile hydrothermal synthesis route. As a photocatalyst, it demonstrates greater than 95% degradation proficiency. Moreover, as an electrocatalyst, NdFeO_3_@MWCNTs shows an overpotential of just 250 mV at 10 mA cm^−2^, a minimum Tafel slope of 67 mV dec^−1^, and an *R*_ct_ of 1.5 Ω. Hence, owing to the dual functionality of the nanocomposite, it can serve as a future photo-electrocatalyst.

## 2. Experimental section

### 2.1. Chemical materials

Neodymium(iii) nitrate hexahydrate (99.5% pure), multi-walled CNTs (99.5% pure), Nafion™ 117, potassium hydroxide (KOH), and sodium hydroxide (NaOH) pellets (98% pure) were procured from Sigma-Aldrich. Analytical grade chemicals like H_2_SO_4_, H_2_O_2_, HNO_3_ and ethanol of 99% purity were used. Iron nitrate (Fe(NO_3_)_3_·9H_2_O) purchased from DUKSAN was utilized. All the chemicals used were of high-grade purity and were used without any supplemental refining steps.

### 2.2. Synthesis of neodymium ferrite (NdFeO_3_)

3 g of Nd(NO_3_)_3_·6H_2_O and 4.5 g of Fe(NO_3_)_3_·9H_2_O were dissolved in deionized water. Both solutions were subjected to vigorous shaking, and their pH was maintained around 9–10 by the dropwise addition of 3 M NaOH. After mixing, the solution was stirred for 30 minutes. Hydrothermal approach have been adopted for material designing. The solution from the beaker was transferred into a Teflon vessel and filled up to 60–70% of its volume. The Teflon-lined autoclave was placed in a heating oven and was subjected to heating for 6 hours at 160 °C. The sample was dried at 70 °C after neutralization and washing, and a powdered mixture was acquired after grinding. The powdered sample was preserved for further characterization.

### 2.3. Functionalization of MWCNTs

Functionalization of MWCNTs is a vital step in the entire nanocomposite design process. The multi-walled CNTs were treated with a mixture of acids (H_2_SO_4_ and HNO_3_) and H_2_O_2_. The MWCNTs were left in these acids for a few hours at 120 °C. Then, after activation, the nanotubes were neutralized by continuous washing. The nanotubes were filtered and dried in a vacuum oven at 70 °C. Afterwards, the nanotube material was ground, heated, and stored for use after adequate drying.

### 2.4. Synthesis of NdFeO_3_-doped MWCNTs

Initially, 100 mg of functionalized MWCNTs was dispersed in 40 mL of deionized water under continuous stirring. Subsequently, 20 mg of the NdFeO_3_ precursor was added to the dispersion, corresponding to an NdFeO_3_ : MWCNTs mass ratio of 1 : 5. The resulting composite therefore contained approximately 16.67 wt% NdFeO_3_ and 83.33 wt% MWCNTs. The pH was adjusted to approximately 10, and the mixture was continuously stirred for 30 min. The resulting suspension was then transferred to a Teflon-lined autoclave and hydrothermally treated at 160 °C for 6 h. After completion of the reaction, the product was neutralized by continuous washing, filtered, dried, ground, and stored for further characterization. The schematic diagram is shown in Fig. 1.

**Fig. 1:**
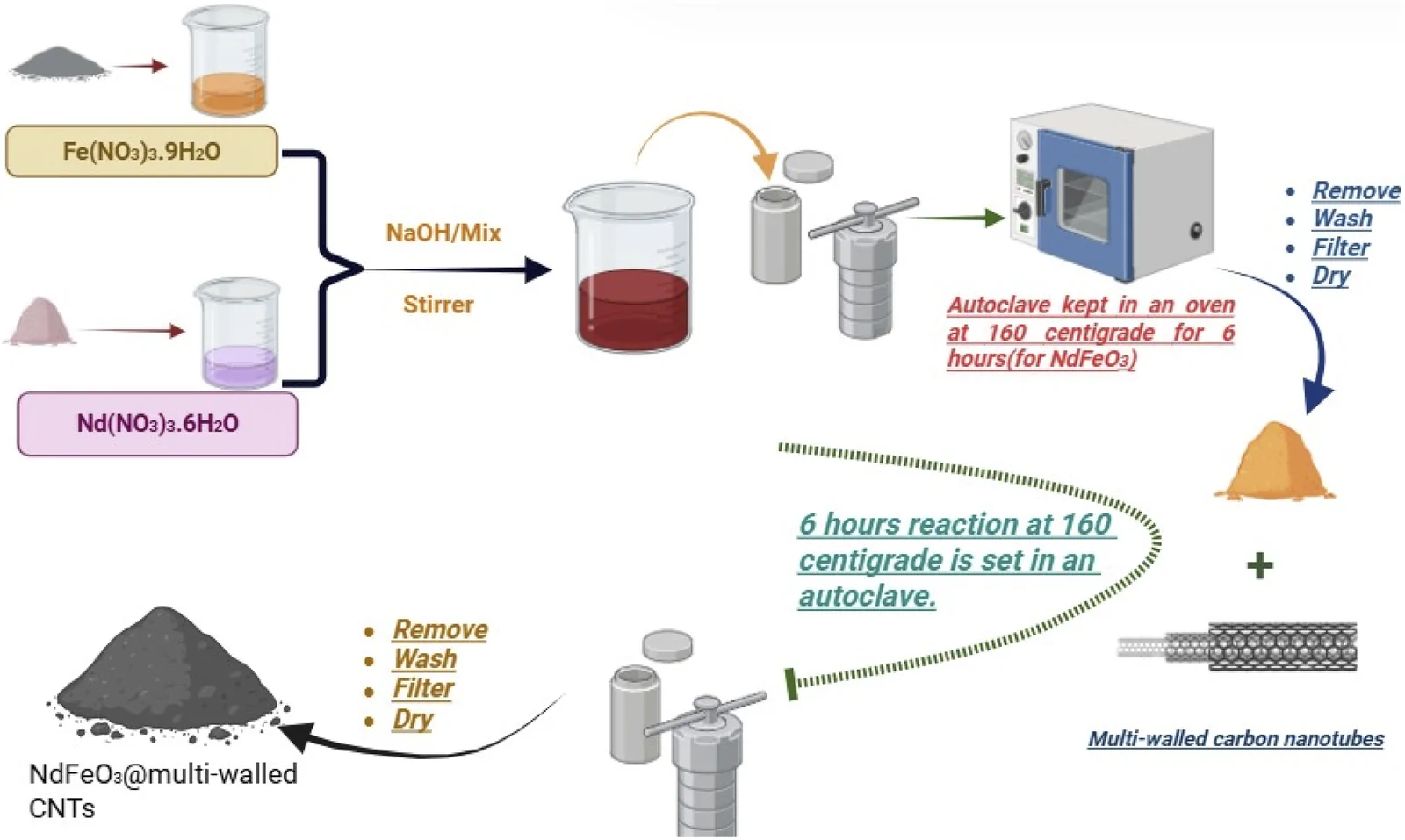
Schematic representation of NdFeO_3_@MWCNTs nanocomposite synthesis.

## 3. Characterization

The prepared nanocomposite material was characterized for structural, morphological, and compositional properties using different characterization techniques. X-ray diffraction was performed using an XRD spectrometer (Rigaku D/max-2500 X-ray diffractometer) for deciphering the phase and structure of the crystal. FTIR (Fourier Transform IR) was employed for detecting multiple functional groups in the compound arising from different bonds stretching and bending vibrations. A PerkinElmer Spectrum IR version 10.6.2 FTIR spectrometer was used for these studies. Morphological interpretation was carried out using a 6490 (LA) scanning electron microscope. An S-200D R & M England UV spectrophotometer was used for studying the photocatalytic degradation efficiency. A transmission electron microscope (JEOL JEM-1230, Akishima, Japan) was used for analysis. A thermogravimetric Q50 V6.2 Build 187 analyzer was employed to conduct TGA measurements (New Castle, DE, USA). The sample was subjected to heating at a rate of 30 °C min^−1^ to 600 °C, with a consistent N_2_ gas flow (20 mL min^−1^). Raman spectra were analyzed using “LabSpec 6” from HORIBA Scientific. Moreover, the absorption as well as bandgap (*E*_g_) studies for the respective material were conducted using a UV-visible spectrophotometer (Shimadzu UV-1800, Japan). The emission peaks corresponding to the transition of electrons in various bands were obtained using a xenon lamp equipped spectrofluorometer (*λ*_ex_ range = 190–880 nm) for photoluminescence (PL) studies.

### 3.1. Photocatalytic experiments

Different amounts of photocatalyst were added to the contaminated solution to evaluate its effectiveness for degradation. At ambient temperature, all factors were examined thoroughly. The contaminated solution was left in the dark for 30 minutes. Numerous parameters, such as catalyst amount, activator induction, pollutant concentration, catalyst reproducibility, and pH, were investigated. H_2_O_2_, an oxidizing agent, was used as the activator. Dilute HCl and NaOH solutions were used to adjust the pH. An S-200D R & M England UV spectrophotometer was used to record the results. Finally, the degradation efficiency was calculated from the following equation:3
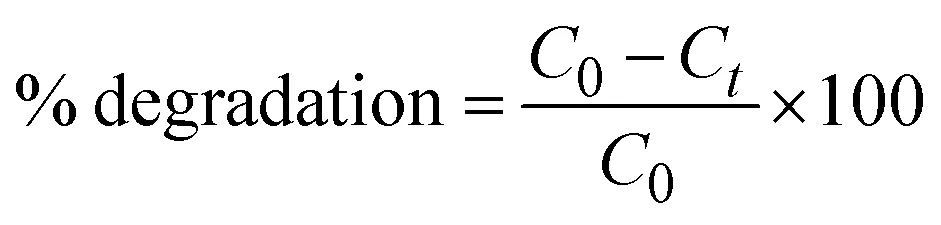
The initial contaminant concentration is *C*_0_, and the final concentration is *C*_*t*_. However, the kinetic studies show a pseudo-first order reaction and is calculated from the following equation:4
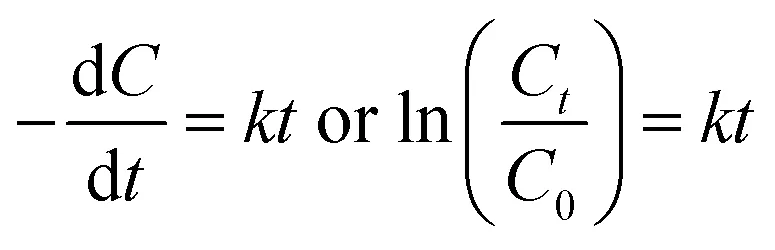


The degradation plot is shown in Fig. 8(a), with *C*_*t*_/*C*_0_ on the *y*-axis and time on the *x*-axis, demonstrating the decrease in pollutant concentration with time.

### 3.2. Electrochemical performance

The electrochemical performance of the electrocatalyst was tested using a Gamry, Interface 1000E workstation, which comprised a three-electrode system. The electrodes included the reference electrode, working electrode, and counter electrode. A 1 M KOH solution served as an electrolyte for the OER. In the current experiment, NdFeO_3_@MWCNTs coated on a nickel foam piece with dimensions of 1 cm × 1.5 cm was employed as the working electrode. Ag/AgCl was the reference electrode, and a platinum wire served as the counter electrode. For electrochemical analysis, linear sweep voltammetry (LSV), cyclic voltammetry (CV), and electrochemical impedance spectroscopy (EIS) experiments were performed in a 1 M KOH solution with a pH of 13. The Nernst equation eqn (5) was used for potential calibration relative to the reversible hydrogen electrode.

5 *E*_(RHE)_ = 0.197 + *E*_(Ag/AgCl)_ + 0.059 × pH

0.197 V is the Ag/AgCl standard electrode potential in eqn (5). Eqn (6) shows the determination of the overpotential (*η*):

6 *η* = *E*_(RHE)_ − 1.23 V

Tafel equation was used for the Tafel slope derivation as shown in eqn (7):

7 *η* = *b* log *j* + *a*

Here, “*a*” is the intercept, “*b*” is the Tafel slope, current density is “*j*”, and *η* is the overpotential. After *C*_dl_ calculation from the slope obtained while plotting the average current density on the *y*-axis and varying the scan rates on the *x*-axis, the electrochemical active surface area (ECSA) is derived from *C*_dl_ as shown in eqn (8):

8 ECSA = *C*_dl_/*C*_s_

“*C*_s_” is the nickel foam specific capacitance, with a value in the range of 0.02–0.08 mF cm^−2^.^37^*C*_s_ is chosen as 0.02 mF cm^−2^ in the current experiment.

### 3.3. Mechanism of dye degradation

Degradation occurs in four crucial steps with the excitation of the nanocomposite material being the first step.

#### 3.3.1. Absorption of light and electron excitation

The nanocomposite precursor (NdFeO_3_) absorbs photons with energy equal to or greater than its band gap to eject electrons from its valence band to the conduction band, leaving holes in the valence band of the material, as shown in the proposed eqn (9):

9 NdFeO_3_ + *hν* → e_CB_^−^ + h_VB_^+^

#### 3.3.2. Transfer of electrons to MWCNTs

MWCNTs provide an extensive conductive network due to their low Fermi level. Hence, the electrons remain in the conduction band, thereby lessening the intensity of electron–hole recombination. Hence, the degradation efficiency increases in this way, as shown in eqn (10):

10 e_CB(NdFeO_3_)_^−^ → MWCNTs

#### 3.3.3. Generation of possible reactive O_2_ species (ROS)

Superoxide radicals (˙O_2_^−^) formed in eqn (11) and hydroxyl radicals (˙OH) formed in eqn (12) and (13) are reactive O_2_ species. Superoxide radicals are probably generated when dissolved oxygen reacts with the accumulated electrons on MWCNTs. On the other hand, holes in the valence band oxidize water and hydroxide ions to form hydroxyl radicals.

11 O_2_ + e^−^ → ˙O_2_^−^

12 H_2_O + h^+^ → ˙OH + H^+^

13 OH^−^ + h^+^ → ˙OH

#### 3.3.4. Dye degradation

ROS, due to their robust oxidative strength, carry out the cleavage of dye chromophoric groups (color-imparting groups) and dye mineralization into possible less-pernicious or non-pernicious components, such as CO_2_ and H_2_O, as shown in eqn (14). Some inorganic substances are also generated during the mineralization process.

14 Dye + ˙OH/˙O_2_^−^ → intermediate products → (CO_2_ + H_2_O)

To further illustrate the proposed charge-transfer pathway, the band alignment and the possible mechanism of reactive species generation in the NdFeO_3_@MWCNTs nanocomposite are presented in Fig. S9. The schematic demonstrates photo-induced electron–hole generation in NdFeO_3_, electron migration through the conductive MWCNT framework, and the subsequent formation of reactive oxygen species responsible for Victoria blue B degradation.^38^

## 4. Results and discussions

Characterization techniques, including photoluminescence (PL)/UV-vis spectroscopy, XRD, FTIR spectroscopy, TGA, TEM, RAMAN, BET, SEM, and EDX spectroscopy have been employed in the best way to investigate the photocatalyst's light-capturing capabilities, structural parameters, functional groups, morphology and elemental composition. All these approaches are discussed in detail in the following paragraphs.

### 4.1. PL/UV-visible spectrophotometric study

Photoluminescence (PL) technique is used to obtain information regarding the efficiency of photocatalytic materials in isolating charge carriers. The photogenerated carriers, such as holes and e^−^, tend to recombine, thereby decreasing the degradation proficiency. Hence, the PL/UV-vis spectrum signifies this aspect, as shown in Fig. 2(a), which indicates a lower e^−^–hole recombination tendency, manifesting a greater engagement time in the degradation phenomenon. Moreover, advancing research reflects the concept of relating these emissions to the material's interfacial properties.

**Fig. 2:**
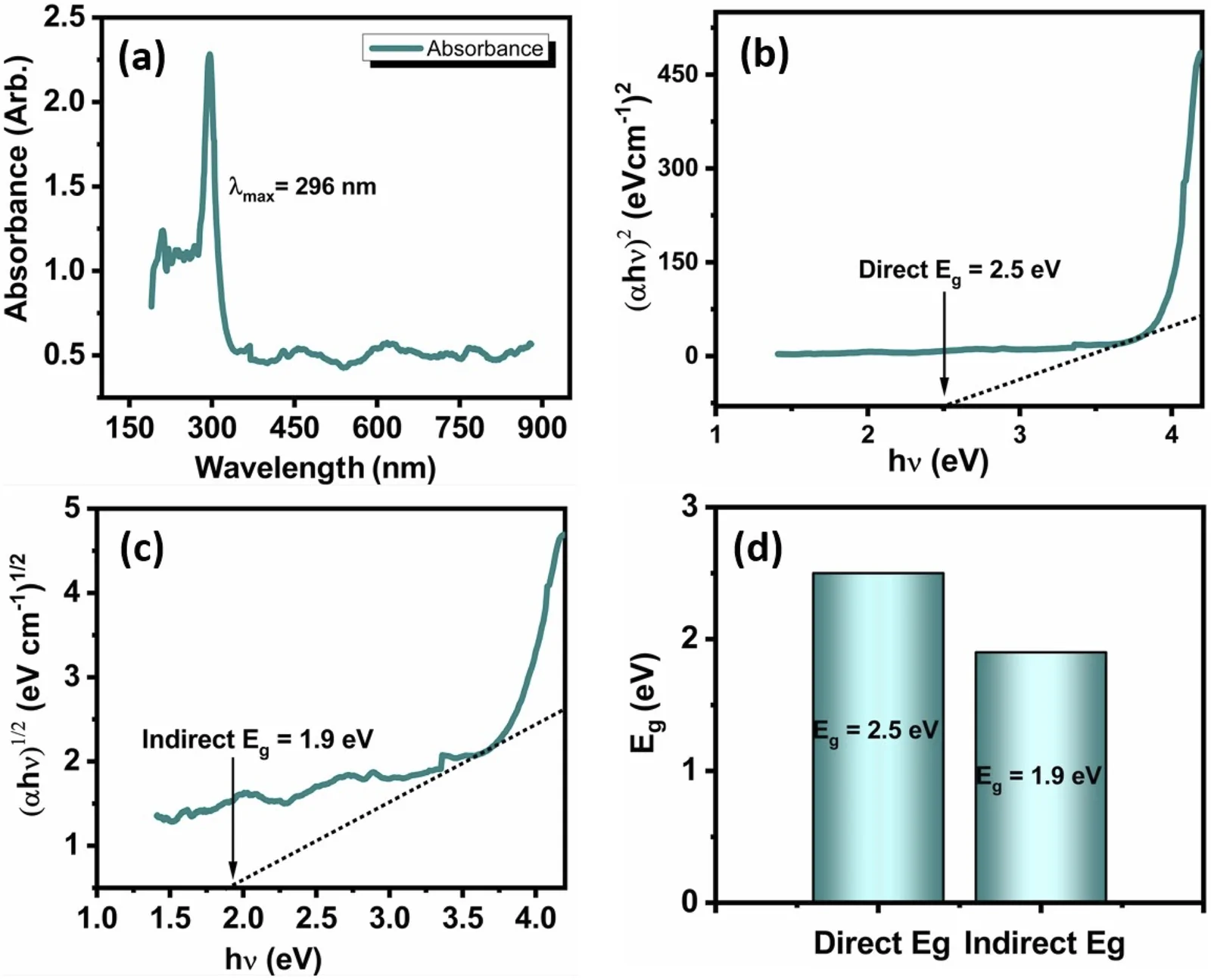
(a) Peak showing the *λ*_max_ for NdFeO_3_@MWCNTs. (b) Direct band gap energy from the Tauc plot. (c) Indirect band gap energy. (d) Comparison of direct and indirect band gap energies.

Our designed nanostructured material, NdFeO_3_@MWCNTs, was tested for its ability to harvest UV-visible light. The compound was tested using a UV-visible spectrophotometer. The results showed the robustness of the nanocomposite in capturing light in the 200–800 nm wavelength range. This reflects the effectiveness of the photocatalyst in the UV and visible regions. The band-gap energy is determined using the Tauc plot.^39^ The equation below provides the parameters for plotting the graph:

15 (*αhν*)^2/*n*^ = *A*(*hν* − *E*_g_)

Here, the photon energy is connoted by “*hν*”, “*h*” signifies Planck's constant and *h* = 6.63 × 10^−34^ J s, and “*α*” is the absorption coefficient. The optical band-gap energy is denoted by “*E*_g_”, “*ν*” is the frequency, and “*A*” is a constant, which is material-dependent. Hence, the direct band gap of the synthesized NdFeO_3_@MWCNTs is 2.5 eV. Fig. 2(b) shows the light-harvesting potency of the photocatalyst in the visible range.

### 4.2. FTIR studies

FTIR studies of NdFeO_3_@MWCNTs reveal its structural characteristics, types of representative functional groups, and molecular arrangements. FTIR was conducted from 400 to 4000 cm^−1^ under normal room conditions. Multiple metal–oxide vibrations in the ABO_3_-type NdFeO_3_ perovskite match the absorption bands that exist at different wavenumbers. Absorption signals of Fe–O appear at 630 cm^−1^. The absorption peak at 936 cm^−1^ is due to NO_3_^−^ ^40^ as nitric acid has been used for the functionalization of MWCNTs. Rare earth metal oxide peaks appearing at higher wavenumber have been reported in the literature, and thus, the peaks appearing at around 794 cm^−1^ have been attributed to the stretching vibration of neodymium oxides, such as Nd–O or Nd–O–Nd. Furthermore, the peak at 891 cm^−1^ is attributed to C–H out-of-plane bending,^41^ which appears potentially due to the presence of organic compounds in the MWCNTs. The bands shift to shorter wavenumbers upon doping with MWCNTs. Due to the functionalized MWCNTs, the signal at 1406 cm^−1^ appears due to C–O stretching owing to carbonate symmetric stretching. The peak at 1538 cm^−1^ is due to C=C stretching vibrations due to the presence of MWCNTs.^42^ The O–H stretching band observed at 3694 cm^−1^, together with the corresponding O–H bending vibration, can be attributed to adsorbed H_2_O molecules. The O–Fe–O stretching signal appears at 1030 cm^−1^. The broad peak at 3149 cm^−1^ is due to the O–H bond of the acidic –COOH groups introduced through the acid treatment of MWCNTs. Furthermore, the decrease in the intensity of the bands and the peak shifts towards some higher wavenumbers confirm the successful incorporation of the modified MWCNTs in the precursor NdFeO_3_.

### 4.3. XRD studies

X-ray diffraction (XRD), undoubtedly, is a convincing technique to investigate the crystallinity, crystal size, and constructional irregularities within nanostructured materials and is used for decoding the exceptional structural aspects of NdFeO_3_@MWCNTs. Prominent separate intense peaks are the acid test for the presence of crystalline nanocomposites. Apart from the crystalline aspects, XRD analysis provides information about the phase refinement and diffraction pattern of multi-walled CNTs and NdFeO_3_ and is shown in Fig. 6. First, the peaks at 2*θ* = 16.16°, 21.52°, 29.05°, 32.41°, 33.40°, 34.91°, 36.93°, 40.61°, 49.83°, 51.83°, 53.51°, and 57.21° with Miller indices (*hkl*) of (121), (022), (040), (042), (233), (242), (143), (044), (444), (354), (172), and (165), respectively, signify the diffraction pattern of NdFeO_3_, resembling ICDD-JCPDS #96-222-3289 and are denoted by green lines in Fig. 3(b). The peaks at 2*θ* = 27.8°, 43.30°, and 59.21° with *hkl* (002), (101), and (103), respectively, closely match card ICDD-JCPDS #96-101-1061 and are attributed to MWCNTs. This is indicated by orange lines in Fig. 3(b). Moreover, the crystallographic parameters prove the cubic crystal system of NdFeO_3_ according to the COD database file of JCPDS card number 96-222-3289. Apart from this, diffraction data are utilized for deriving the crystallite size using the Debye–Scherrer formula as shown below:16
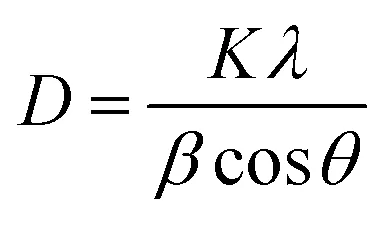
Here, *D* shows “mean crystalline size”, *K* is the Scherrer constant, whose value equals 1 for spherical-shaped crystallites, *λ*(Cu Kα) = 0.154 nm, *β* = full width at half maximum (FWHM), and *θ* signifies the Bragg angle. Hence, taking advantage of the Scherrer formula, the average crystallite size is 16.23 nm. Furthermore, the absence of any auxiliary peaks serves as a litmus test of the synthesis of proposed NdFeO_3_@MWCNTs.

**Fig. 3:**
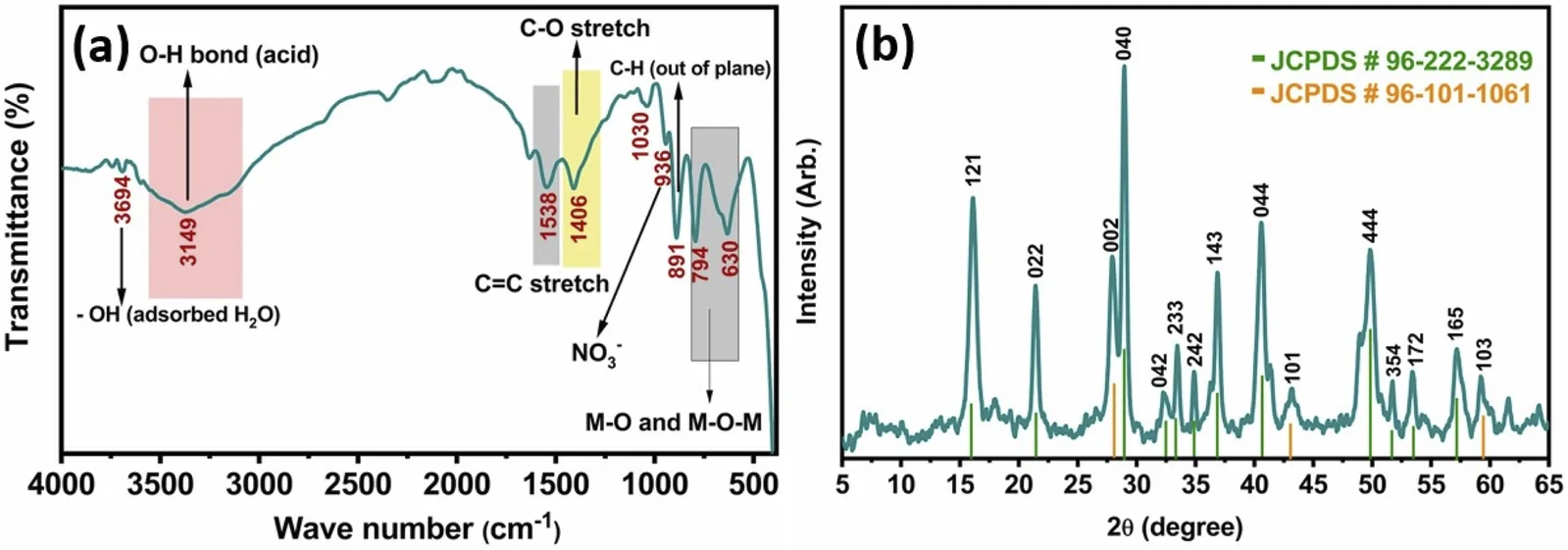
(a) FTIR spectrum of NdFeO_3_@MWCNTs. (b) XRD pattern of NdFeO_3_@MWCNTs.

### 4.4. Raman analysis

The Raman spectrum shown in Fig. 4(a) presents evident vibrational peaks corresponding to MWCNTs, as well as the rare earth metal-based perovskite oxide material. Definite A_g_ and B_3g_ modes are assigned to phonon emissions in the low-wavenumber domain and are attributed to the NdFeO_3_ oxide precursor. However, the successful incorporation of MWCNTs is reflected in the respective D and G bands.^43^ The D band at around 1338 cm^−1^ (ref. 44) and the G band at about 1578 cm^−1^ (ref. 45) are the indicators of the intensity of defects as well as the graphitization (sp^2^-hybridized carbon). Moreover, the *I*_D_/*I*_G_ ratio indicating the defect intensity is 2.1 in the current case, demonstrating that the increased defects might also be due to defects incurred during MWCNT functionalization with acids to some extent. MWCNTs are not a single-layered structure; rather, they are multi-layered, and this CNT multilayer structure is evidenced by the D band presence around 2700 cm^−1^, as also reported in a previous study.^46^

**Fig. 4:**
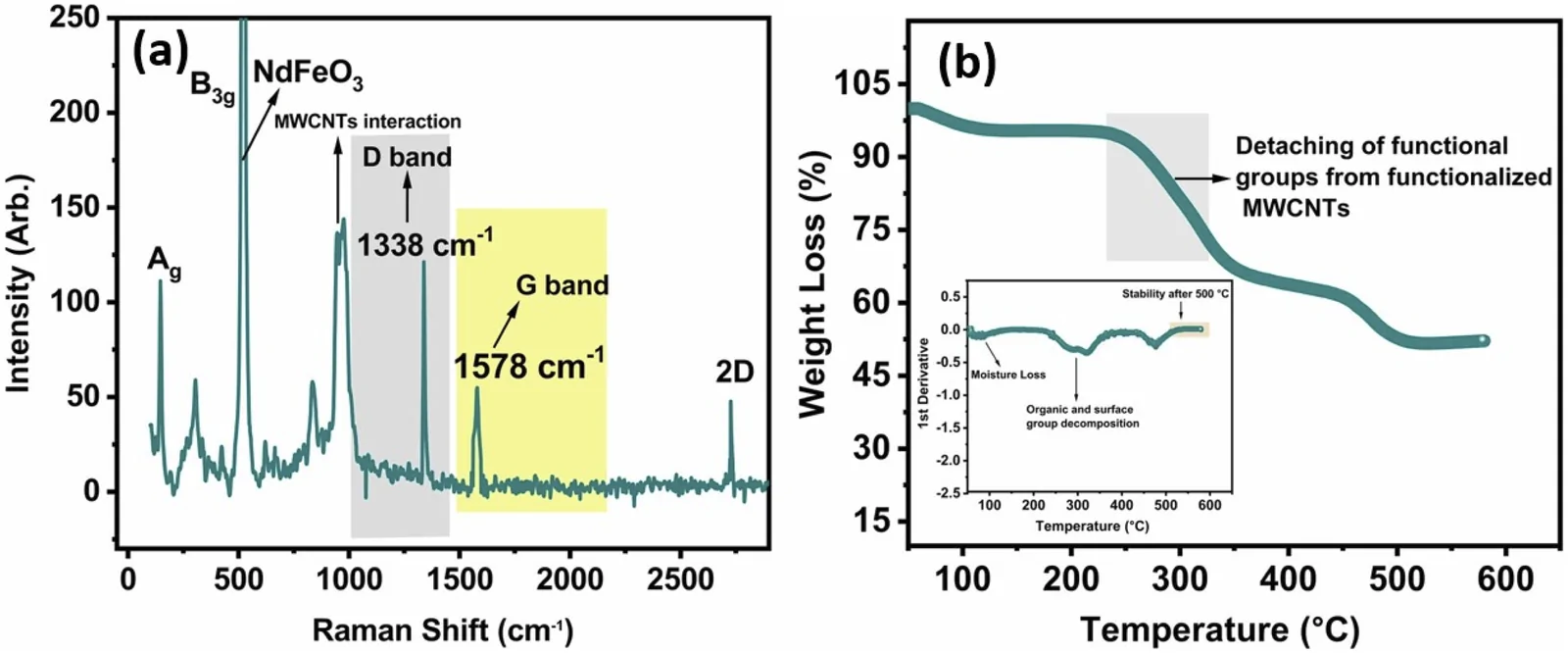
(a) Clearly evident D and G bands are highlighted around 1350 cm^−1^ and 1580 cm^−1^, respectively, due to MWCNTs. (b) Material stability as depicted from the TGA graph, with the inset showing DTG.

Hence, the Raman spectrum gives a prominent account of NdFeO_3_@MWCNTs formation. The shift in intensity and wavenumber is due to the interfacial reciprocity between the precursor and the multi-layered CNTs.

### 4.5. Thermogravimetric analysis

TGA provides productive information regarding the material's stability by showing weight loss with increasing temperature. The technique assists in the evaluation of the thermal stability of both carbon- and non-carbon-based materials. In the case of NdFeO_3_@MWCNTs, the weight loss below 100 °C is due to adsorbed H_2_O or moisture, indicating the hydrophilicity of the composite surface. However, in the first-derivative DTG curve, around 300 °C, the weight loss is due to the detachment of the functional group (COOH owing to functionalized CNTs). Afterward, the NdFeO_3_@MWCNTs stability is reflected in the negligible weight loss with the increase in temperature beyond 500 °C, which is due to the NdFeO_3_ precursor framework. Moreover, a sharp loss followed by a subsequent stable phase at an intensely high temperature can be seen in the DTG curve in Fig. 4(b) inset. Hence, the interpretation confirms the integrity of NdFeO_3_@MWCNTs and the potential application of the nanocomposite as a high-temperature material.

### 4.6. Surface morphological studies

Scanning electron microscopy (SEM) is exploited as a viable technique for the topographical interpretation of nanomaterials. Along with morphological characterization, SEM is used for imaging purposes. Hence, the SEM profile elucidates the asymmetrical stacking of particles in the NdFeO_3_@MWCNTs nanocomposite. The elongated lumps in the morphology of the NdFeO_3_ material and the networked web of MWCNTs are evident from the SEM image taken at 300 nm, as shown in Fig. 5(c). The particles are haphazardly distributed over the tube-like nanostructures. This points to the delicately fabricated NdFeO_3_@MWCNTs structure as shown in Fig. 5(c). Magnified images disclose a tubular structure; hence, differentiation becomes simplistic as the magnification increases. Furthermore, the presence of compact packing is attributed to ferrites. Ferrites have a magnetic nature, and the attraction among particles incites particles to associate, resulting in aggregation.

**Fig. 5:**
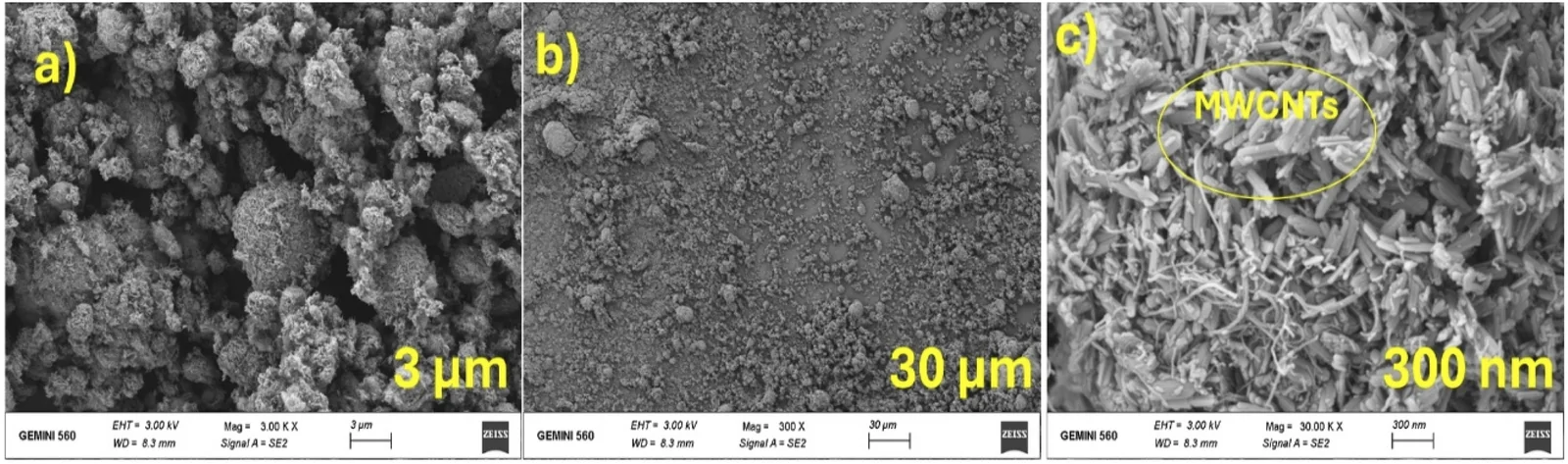
SEM images taken at different magnifications: (a) 3 µm, (b) 30 µm, and (c) 300 nm.

### 4.7. EDS studies

Energy dispersive X-ray spectroscopy (EDS) is a technique utilized with SEM to provide sample mapping. EDS is an elemental analysis tool for determining composition by weight and atomic proportion. The EDS profile of the material in the study displays the presence of Nd, Fe, C, and oxygen. The prominent peaks corresponding to neodymium, oxygen, iron, and carbon in the EDS spectra are indicators of their elemental composition. The experimental results show the presence of Nd, Fe, O, and C in 14.5%, 13.2%, 26.2%, and 46%, respectively, as shown in Fig. 6.

**Fig. 6:**
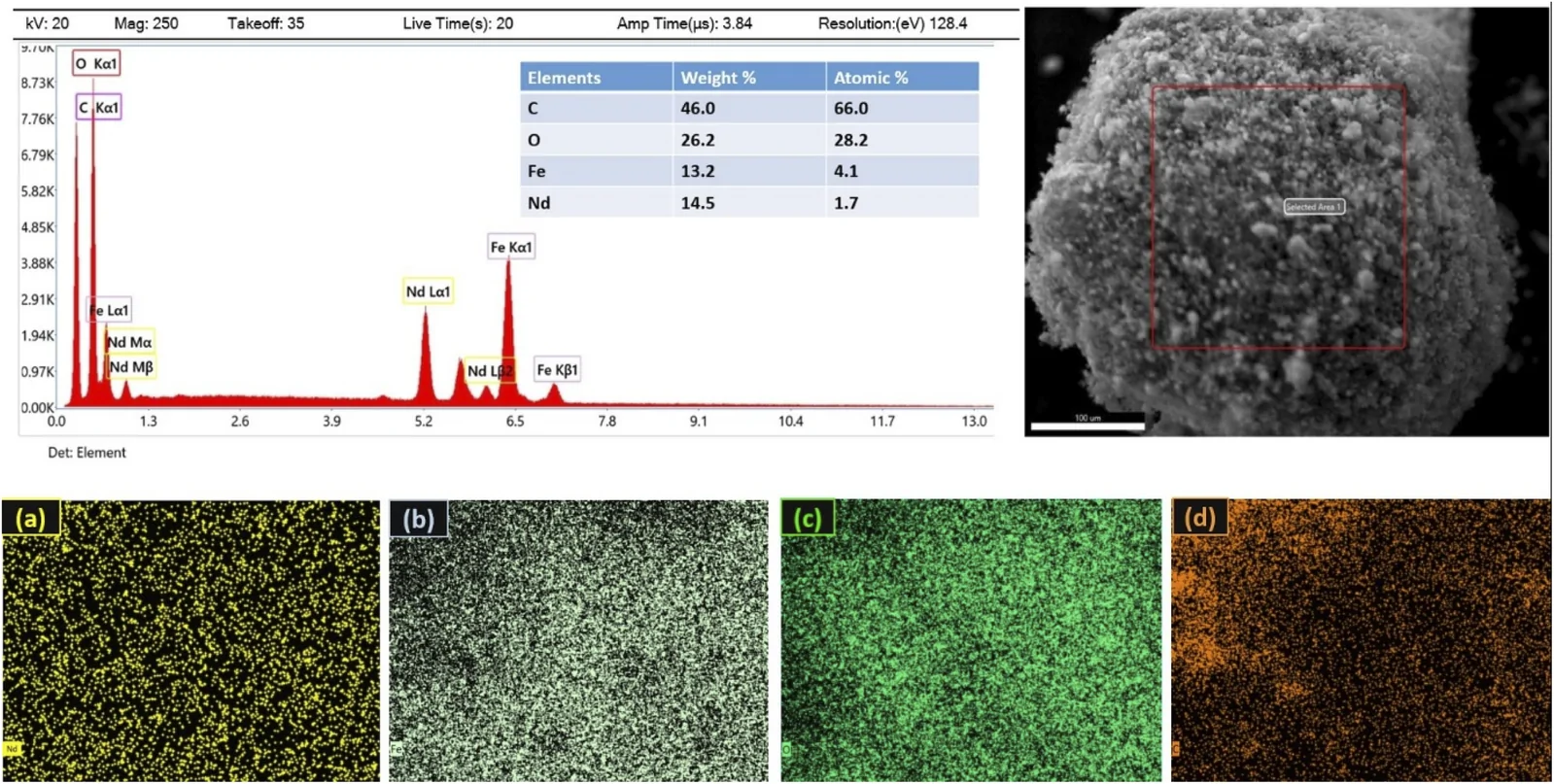
EDX mapping with elemental percentages. (a) Neodymium (yellow). (b) Iron (greenish granite). (c) Oxygen (green). (d) Carbon (orange).

### 4.8. TEM profiling

Transmission electron microscopy (TEM) reveals a great sum of probed enriched information about the NdFeO_3_@MWCNTs nanocomposite at an atomic level. Fig. 7 accounts for two well-defined nano-level structures. The brighter area containing an intertwined network and elongated tubes of varying dimensions is composed of multi-walled CNTs (MWCNTs), while the darker area comprises of NdFeO_3_ crystals. The NdFeO_3_ crystal nanostructure is randomly stacked, as shown in Fig. 7. Moreover, the aggregated NdFeO_3_ nanostructure as observed in TEM is in agreement with the closely stacked structure as evident from SEM profiling, which is attributed to the magnetic nature of ferrites. This confirms the successful formation of NdFeO_3_. The incorporation of the entire precursor over the tubular structure of the carbon nanotubes in NdFeO_3_@MWCNTs can be visualized in the figure, where NdFeO_3_ particles are stacked over nanotubes. Depending upon the synthesis scheme, the MWCNTs vary in dimension from 1.85 nm to 7.53 nm, as elucidated through Digital Micrograph software, as shown in Fig. 7. The crystalline parameter of the nanocomposite is revealed from the Fast Fourier Transform (FFT) as well as the inverse FFT (IFFT), showing concentric rings in Fig. S4 in the SI. In IFFT images, the parallel concentric lattice fringe pattern is due to the graphitic structure of MWCNTs.

**Fig. 7:**
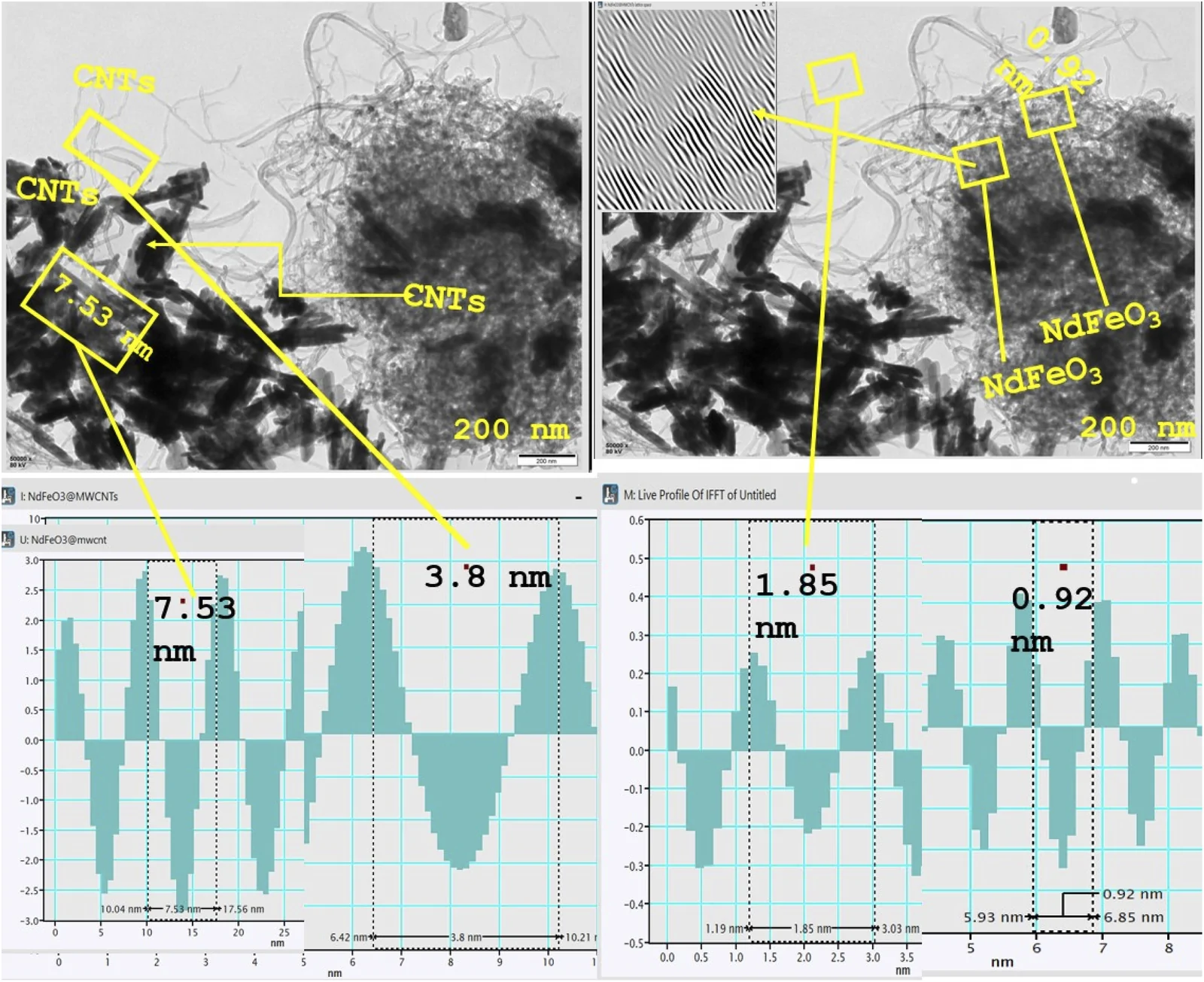
TEM profile of the perovskite NdFeO_3_@MWCNTs nanocomposite (top row) with an inset showing the lattice fringes, demonstrating the morphological texture of multi-walled carbon nanotubes (MWCNTs) and aggregated stacked NdFeO_3_ nanoparticles. The bottom row presents the corresponding line profile analyses, indicating various diameters of NdFeO_3_ as well as MWCNTs (0.92 nm for NdFeO_3_ and 7.53 nm, 3.8 nm, and 1.85 nm for CNTs).

### 4.9. BET (Brunauer–Emmett–Teller) analysis

Nitrogen adsorption–desorption analysis was performed to investigate the surface characteristics and pore structure of the NdFeO_3_@MWCNTs nanocomposite. As presented in Fig. S8(a), the adsorption–desorption isotherm exhibits a typical type IV isotherm with a hysteresis loop, indicating the presence of mesoporous characteristics within the composite structure.^47^ The BET surface area of the prepared NdFeO_3_@MWCNTs nanocomposite was calculated to be 31.638 m^2^ g^−1^, with a total pore volume of 0.352 cm^3^ g^−1^. Furthermore, BJH pore size distribution analysis (Fig. S8(b)) revealed an average pore diameter of approximately 3.49 nm, confirming the formation of a mesoporous framework.

Although the obtained surface area is moderate compared with some highly porous carbon-based materials, it remains sufficient for photocatalytic applications due to the synergistic role of the MWCNT network and NdFeO_3_ nanoparticles. The relatively lower surface area can be attributed to the incorporation of NdFeO_3_ nanoparticles onto the MWCNT framework, which may partially occupy or block the accessible nanotube surface and pore channels. Additionally, the magnetic nature of ferrite nanoparticles can promote particle aggregation, reducing the accessible surface area. However, the intimate interaction between NdFeO_3_ and conductive MWCNTs provides efficient charge migration pathways, suppresses electron–hole recombination, and enhances the photocatalytic performance beyond the contribution of surface area alone.

The enhanced multifunctional catalytic performance of NdFeO_3_@MWCNTs can be attributed to the synergistic interaction between the NdFeO_3_ perovskite phase and the conductive MWCNT network. The crystalline NdFeO_3_ provides abundant catalytic active sites, while the MWCNT framework improves electrical conductivity and serves as an efficient pathway for charge migration. The intimate interface between NdFeO_3_ and MWCNTs suppresses electron–hole recombination during photocatalysis and facilitates interfacial charge transfer during the OER. Moreover, the mesoporous characteristics and increased accessible surface area promote reactant interaction and provide additional active sites. These combined structural and electronic advantages contribute to the enhanced photocatalytic degradation of Victoria blue B and improved oxygen evolution activity.

### 4.10. Photocatalytic degradation

#### 4.10.1. Catalyst dosage effect

Catalyst dosage shows a proportional effect with the increase in catalyst amount. As the amount of catalyst employed increases while keeping the pollutant concentration and pH constant, the time taken by the catalyst to degrade the pollutant is reduced. A 97% efficiency of pollutant degradation is achieved with a 5 mg catalyst dose, and the efficacy was about 78% with 1 mg catalyst as the minimum dose. The effect of catalyst dosage on the pollutant-removal efficiency is best illustrated in the graph given in Fig. S5 in the SI. All the data were collected at a wavelength of 593 nm, which is considered the optimum wavelength for the degradation study of Victoria blue B according to the literature.^48^

#### 4.10.2. Pollutant concentration effect

The degradation phenomenon slows down as the pollutant concentration increases. A 1000 ppm dye solution takes a comparatively greater time for degradation than a 250 ppm pollutant solution. The degradation of the 1000 ppm solution is slow because of the enrichment of pollutants and the overall decreasing catalytic site availability for the photo-oxidation of the dye molecules. The bar graph in Fig. S6 elucidates the percentage degradation, which clearly shows the removal rate for different concentrations of the dye in a specific timeframe. For the 250 ppm pollutant solution, 97% removal is obtained within the first 5 minutes compared to the 1000 ppm solution, which requires 20 minutes for achieving almost the same degradation efficiency.

Table S2 shows a comparison of our catalyst with those reported in the literature.

#### 4.10.3. Role of the activator

The activator or the oxidizing agent has an incremental impact on the removal efficiency. A 93.17% removal efficiency is obtained with an addition of 2 mL H_2_O_2_. While without the activator, the percentage efficiency was 77.28%. This efficiency was achieved within the first 15 minutes under UV light. Fig. 8(d) represents the enhanced degradation efficiency with the activator in the form of a bar graph. Hydrogen peroxide is considered the best oxidizing agent, with a minimum quantity resulting in a boost in the degradation.

**Fig. 8:**
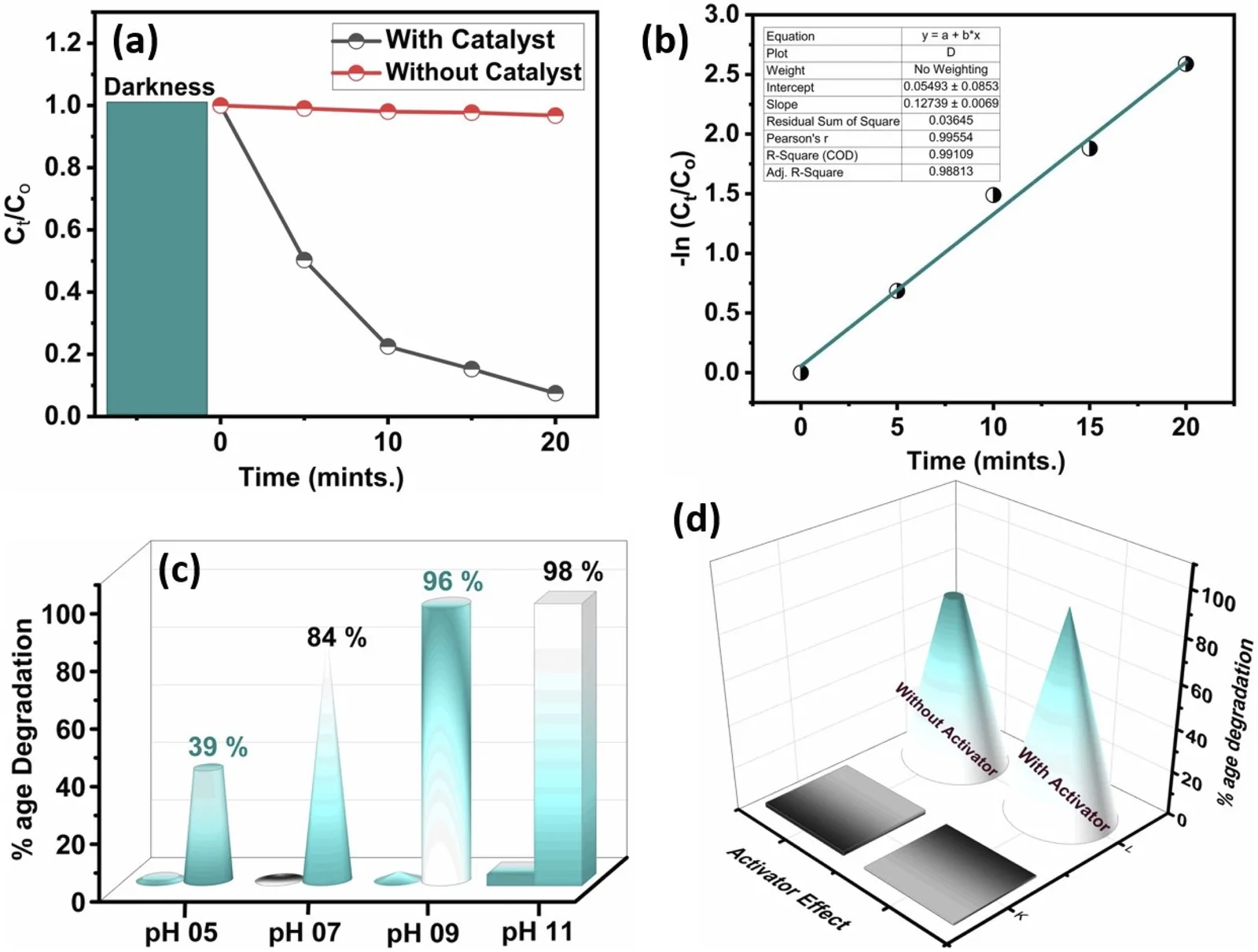
(a) Demonstration of almost negligible or zero degradation without a photocatalyst (straight line). (b) Rate constant defining the pseudo-first order kinetics. (c) Enhanced elimination with the maximum removal at pH 11. (d) Impact of the activator, showing increased degradation.

#### 4.10.4. pH role

The pH also has a significant role to play. The degradation process is fast in a basic medium and slow in an acidic medium at pH = 5. A basic environment is favorable for the photo-driven oxidation process. Moving from acidic to basic environments, the height of the bar graph gives a clear indication of the removal percentage. This is shown in Fig. 8(c), where, with increasing alkalinity, the bar height rises, indicating enhanced efficiency.

### 4.11. Electrochemical studies

All electrochemical measurements for the oxygen evolution reaction (OER) evaluation were performed at room temperature under ambient conditions using a standard three-electrode configuration. The NdFeO_3_@MWCNTs nanocomposite material was tested for its electrochemical parameters through CV, LSV and EIS. LSV is just single potential sweep, either in the forward or in the reverse direction. 1 M KOH solution was used as the electrolyte solution for the OER, and all the electrodes are immersed in the electrolyte. A reverse LSV was performed to avoid the overlap of the nickel foam oxidation peak with the catalyst's oxidation, and the Tafel slope and the overpotential were calculated from it.

The overpotential for NdFeO_3_@MWCNTs was determined from LSV, and the value was 250 mV at a scan rate of 5 mV s^−1^ to achieve a current density of 10 mA cm^−2^. The overpotential increased to about 300 mV for achieving 100 mA cm^−2^ current density, as shown in Fig. 9(d). 10 mA cm^−2^ serves as a benchmark current density, and the performance of all the electrocatalysts is compared at this current density. Moreover, NdFeO_3_@MWCNTs have a very low onset potential of 1.47 V as shown in Fig. 9(a), defining the marvelous oxygen evolution potential of the aforementioned electrocatalyst. To verify the contribution of the catalyst layer, the CV response of the bare Ni foam was compared with that of the NdFeO_3_@MWCNTs-coated Ni foam electrode (Fig. S7). The bare Ni foam exhibited a negligible current response compared with the catalyst-modified electrode, demonstrating that the enhanced electrochemical activity originates from the NdFeO_3_@MWCNTs nanocomposite. Overpotential is the electrocatalyst's intrinsic feature.

**Fig. 9:**
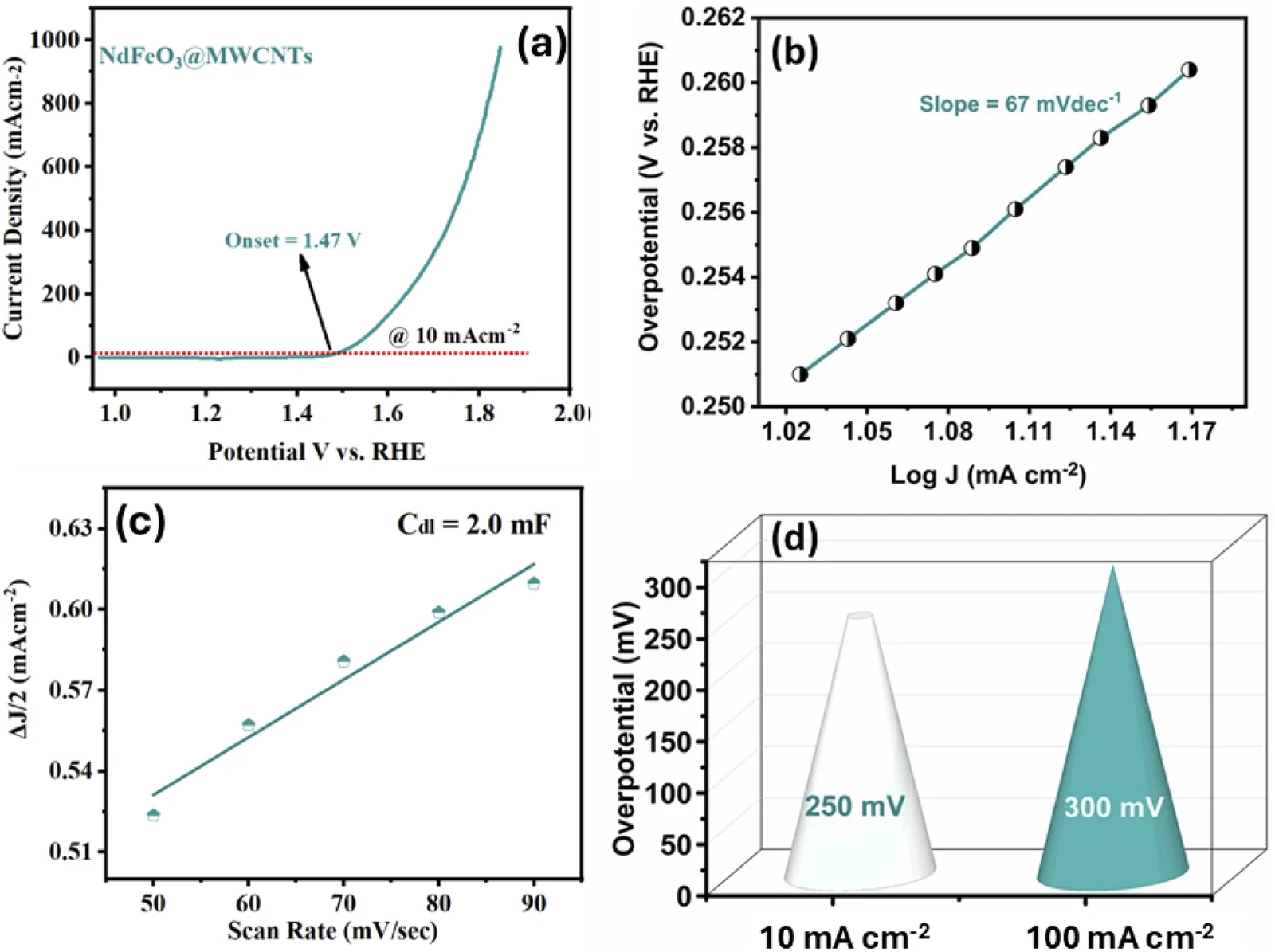
(a) Reverse LSV for NdFeO_3_@MWCNTs with a low onset potential. (b) Tafel plot for NdFeO_3_@MWCNTs. (c) *C*_dl_. (d) Overpotential comparison at 10 mA cm^−2^ and 100 mA cm^−2^.

Oxygen evolution is a more elaborate four-step process and encounters a high energy barrier. Hence, to overcome this energy constraint, an electrocatalyst is employed. The following are the reaction steps^49^ occurring over the surface of an electrocatalyst, eventually leading to O_2_ evolution. OER is a four-electron transport process as shown in eqn (17)–(20) below:

17 H_2_O + * ⇌ OH* + H^+^ + e^−^

18 OH* ⇌ O* + H^+^ + e^−^

OOH* mentioned in eqn (19) is an intermediate, and in the next step, as shown in eqn (20), it leads to O_2_ generation, leaving the active site surface.

19 O* + H_2_O ⇌ OOH* + H^+^ + e^−^

20 OOH* ⇌ O_2_ + H^+^ + e^−^ + *

The currently designed NdFeO_3_@MWCNTs contains a precursor, which is a perovskite structure having an excess of oxygen. There is an alternate path for proceeding this reaction *via* a lattice oxygen-mediated mechanism.^50^ A comparative analysis of the photocatalytic and electrocatalytic performance of the NdFeO_3_@MWCNTs nanocomposite with previously reported materials is provided in Table S1.

The second electrochemical criterion is the Tafel slope, which was found to be 67 mV dec^−1^. The Tafel slope is a fundamental kinetic parameter that can give a clue about the reaction mechanism and the rate-determining step of the oxygen evolution reaction (OER). It is a measure of the overpotential required to raise the current density by a factor of 10. Hence, decreased overpotential in conjunction with a low Tafel slope is a good indicator of NdFeO_3_@MWCNTs' potential for electrochemical applications, such as energy storage and conversion.

With respect to the OER, a smaller Tafel slope is preferred because it reflects more rapid reaction rates and a better electrocatalyst. The observed value of 67 mV dec^−1^ indicates a relatively moderate catalytic activity, which means that the reaction kinetics are hindered by a chemical step after the primary electron transfer. Fig. 9(b) shows the corresponding Tafel plot. Moreover, CV cycles in the non-faradaic region were recorded at different scan rates for the calculation of the double-layer capacitance (*C*_dl_), and then, the electrochemically active surface area (ECSA) was derived from it. ECSA = *C*_dl_/*C*_s_, where *C*_s_ is the specific capacitance for a substrate, which ranges from 0.02 to 0.08 mF cm^−2^. The greater the ECSA, the better the electrocatalyst. The CV cycles in the non-faradaic region are shown in the supplementary data as S2(a), and the *C*_dl_ plot is shown in Fig. 9(c). The *C*_dl_ and ECSA values are 2.0 mF and 100 cm^2^, respectively, for the designed novel NdFeO_3_@MWCNTs nanocomposite material. Fig. 10(a) shows the EIS Nyquist plot with the inset showcasing the CPE circuit. The CPE circuit fits the results well as there is no ideal capacitive behavior; thus, a constant phase element (CPE) is used, where *R*_s_ represents the solution resistance and *R*_ct_ represents the charge transfer resistance of the electrocatalyst. EIS was performed at a DC voltage of 0.6 mV and in the frequency range from 10^5^ to 0.01 Hz. It can be observed from the EIS Nyquist plot semicircle that the synthesized NdFeO_3_@MWCNTs electrocatalyst shows the lowest charge transfer resistance of 1.5 Ω with a minimal solution resistance. The smaller the *R*_ct_, the better is the electrocatalytic behavior. Moreover, the *R*_s_ is 0.4 Ω, which is due to the solution and has a very low value, which is favorable for the electrochemical behavior. Furthermore, the Bode magnitude and phase plots (Fig. S10) were analyzed to obtain additional information regarding the frequency-dependent impedance response and interfacial charge-transfer behavior of the NdFeO_3_@MWCNTs electrode.

**Fig. 10:**
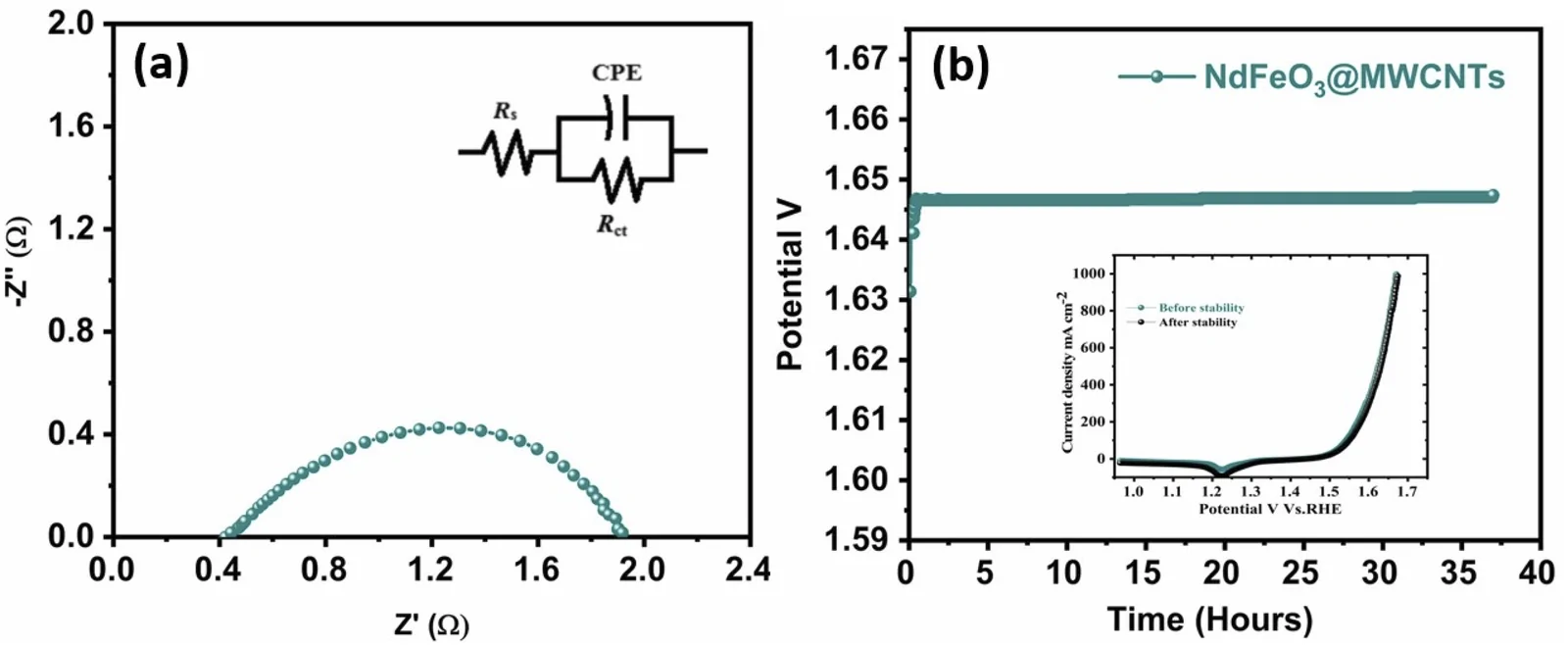
(a) Nyquist plot (giving *R*_s_ and *R*_ct_) with the inset showing the CPE circuit. (b) Extended stability up to 36 hours with the inset showing negligible overpotential change before and after the stability test.

Above all, one more considerable feature of an electrocatalyst is its stability, which is reflected in its durability for industrial applications. Corrosion of the electrocatalyst over time poses challenges. Therefore, Fig. 10(b) illustrates the chronopotentiometric results. Chronopotentiometry was carried out at 50 mA constant current, and the results show the stability of the catalyst even after 36 hours. It supports the use of electrocatalyst for further practical applications. Apart from chronopotentiometric evaluations, the polarization curve in Fig. 10(b) inset demonstrates no or negligible deviation in the overpotential value before and after the stability test.

## 5. Conclusion

In conclusion, the fabricated nanomaterial not only functions as an electrocatalyst but also as a photocatalyst. Key electrochemical features, such as a low Tafel slope, overpotential, and *R*_ct_, are defining parameters in determining the potential of NdFeO_3_@MWCNTs as an electrocatalyst. However, as we are concerned with the photocatalyst behavior, the direct and indirect band gap energies of 2.5 eV and 1.9 eV, respectively, are signatures of extended pollutant oxidation and reduction efficacy. The electrochemical properties such as overpotential (*η*), Tafel slope, electrochemically active surface area (ECSA), and *R*_ct_ were calculated through different tests for the nanocomposite material. Quantifying the outcomes for NdFeO_3_@MWCNTs shows its marvelous OER activity in 1 M alkaline KOH environment, with an *η* of 250 mV at 10 mA cm^−2^ current density, a Tafel slope of 67 mV dec^−1^, an ECSA of 100 cm^2^, and an *R*_ct_ of 1.5 Ω. Additionally, the nanocatalyst demonstrates an excellent degradation efficiency of 97% with just 5 mg catalyst.

The nanocatalyst was prepared through a simple hydrothermal route, and the lower cost of our catalyst than that of noble metal catalysts makes it a worthy choice for use. XRD, FTIR spectroscopy, SEM, TEM, EDX spectroscopy, Raman spectroscopy, and TGA confirmed the synthesis of NdFeO_3_@MWCNTs, as well as the thermo-stability of the catalyst under different temperatures. The successful synthesis of NdFeO_3_@MWCNTs is evidenced by the distinct phonon peaks in the low wavenumber region for the precursor material, alongside the characteristic D and G Raman bands for the CNTs, where the D band confirms their multi-layered nature. In short, the nanocomposite can be employed as an efficient, durable, and facile electrocatalyst for future green energy generation.

## Conflicts of interest

There are no conflicts to declare.

## Supplementary Material

RA-016-D6RA05821C-s001

## Data Availability

Data will be provided upon request. Supplementary information (SI) is available. See DOI: https://doi.org/10.1039/d6ra05821c.
