# Supplementary material for: Lattice oxygen-mediated ABO3-type NdFeO3 perovskite fabricated over multi-walled CNTs for the O2 evolution reaction and photo-induced degradation of Victoria blue B
Source: RSC Adv. 2026 Sep 29;16(45):58346–59. doi: 10.1039/d6ra05821c (PMC13621347; doi:10.1039/d6ra05821c)
Supplement: Supplementary file 1 [file RA-016-D6RA05821C-s001.pdf]

Supplementary Information (SI) for RSC Advances.
This journal is © The Royal Society of Chemistry

Lattice Oxygen Mediated ABO_3-type of $NdFeO_3$ perovskite fabricated over multi-walled CNTs for O_2 evolution reaction and photo-induced degradation of Victoria Blue B.

Rabbia Shoukat [a,#], Sadam Hussain [b,#], Sadia Ata [*,a], Hanaa A. Galeb [c], Aliya Kouser[d], Ayesha Sadiqa[e], Adnan Ashraf[e], Awais Ahmad[e,f*]

[a]School of Chemistry, University of the Punjab, Lahore, 54000, Pakistan;

[b]School of Natural Sciences (SNS), Department of Chemistry, NUST, H12, Islamabad;

[c]Department of Chemistry, Faculty of Science and Arts, King Abdulaziz University, Rabigh, Saudi Arabia

[d]Institute of Chemistry, Khwaja Fareed University of Engineering and Information Technology, Rahim yar khan, Pakistan.

[e]Department of Chemistry, The University of Lahore, Lahore, Pakistan.

[f]Faculty of Engineering and Technology, Shinawatra University, Sam Khok, Thailand

# Equal authorship

*** Corresponding author**

Fabrication of electrocatalyst over Nickel foam piece

$NdFeO_3$@MWCNTs nanocomposite is deposited over nickel foam piece (1 cm x 1.5 cm dimension) with the help of Nafion binder. First, catalyst ink is prepared. Catalyst slurry or ink is prepared from 5 mg sample powder, 2 to 3 drops of ethanol, and 1 drop of Nafion binder. The mixture is mixed and then with the help of micropipette is sprayed over the nickel foam piece.

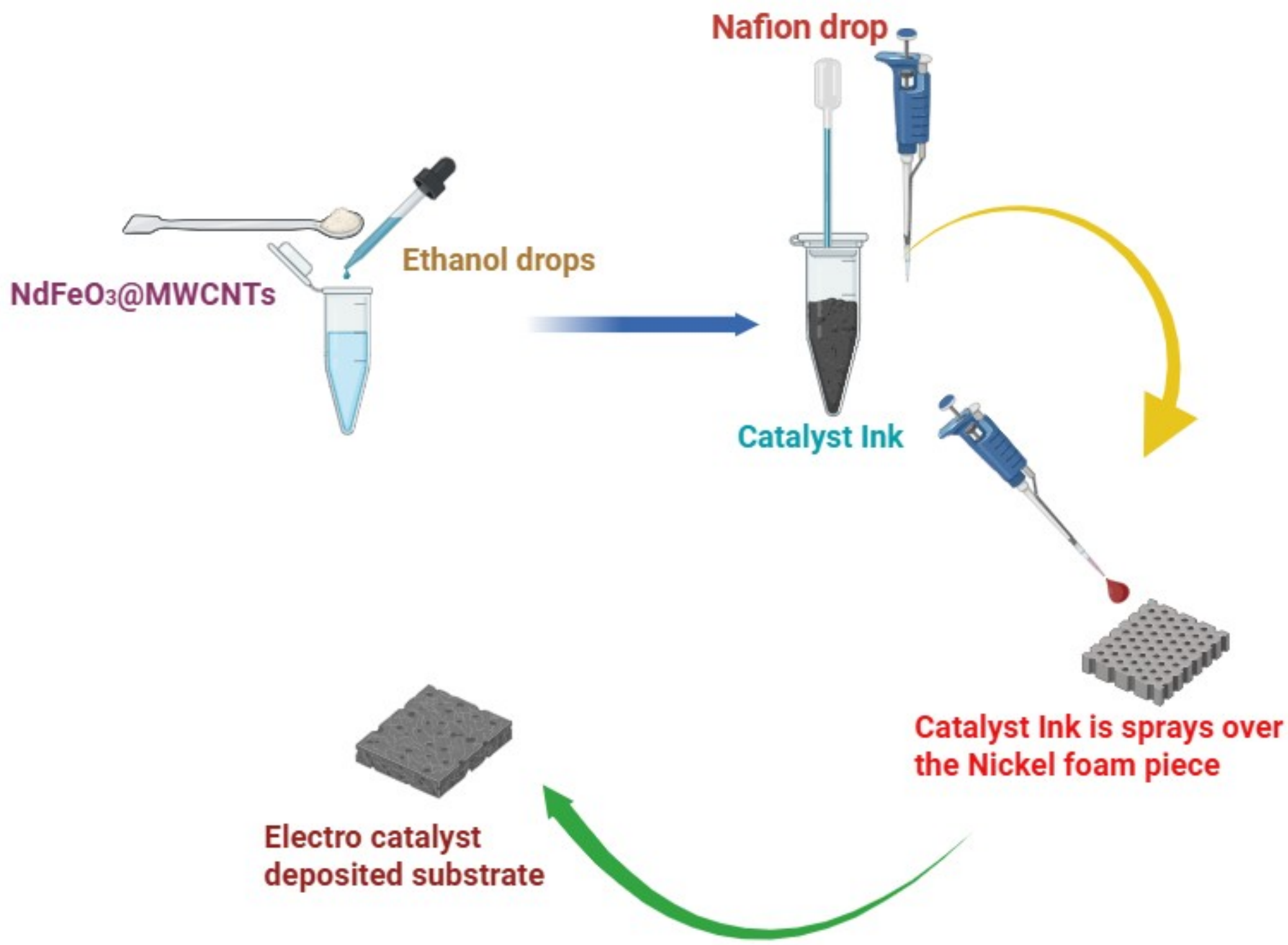

Figure S1. Fabrication of electrocatalyst over Nickel foam

Table S1. Comparison of our catalyst with those of strikingly resembling reported electrocatalysts in literature

Sr. No.	Electrocatalyst material	Overpotential (mV) at 10 $mAcm^{-2}$	Tafel Slope (mV/dec)	Electrolyte	Reference
01.	MWNT-PyPBI-NiCo3-xO4	310 mV	42 mV/dec	1 M KOH	[1]
02.	"Pristine multi-walled carbon nanotubes" in MnO_2-multiwalled carbon nanotubes	575 mV	184 mV/dec	1 M KOH	[2]
03.	$NdFeO_3$/gCN	420 @ 10$mAcm^{-2}$	96mV/dec	1 M KOH	[3]
04	$Nd_{0.9}FeO_3$ (denoted NF10)	370 mV	79	1 M KOH	[4]
05.	Sulphur-incorporated $CoFe_2O_4$/multi-walled carbon nanotubes	360 mV	43 mV/dec	1 M KOH	[5]
06	CoFe@NCNT	340	146.3	0.1 M KOH	[6]
07	IrO_2	364	87.8	1 M KOH	[7]
08.	$NdFeO_3$@MWCNTs	250 mV	67 mV/dec	1 M KOH	This paper

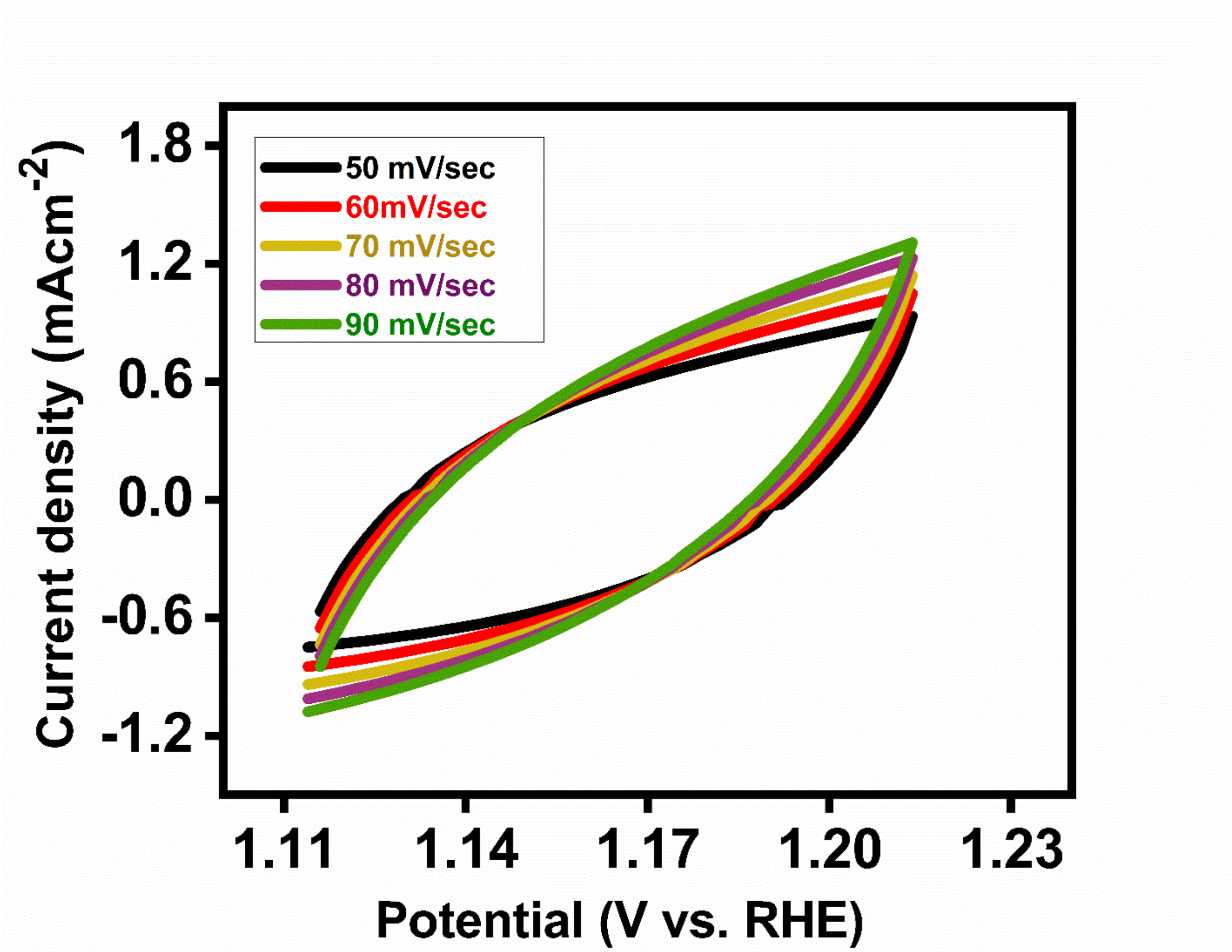

Figure S2. CV cycles at altering scan rates.

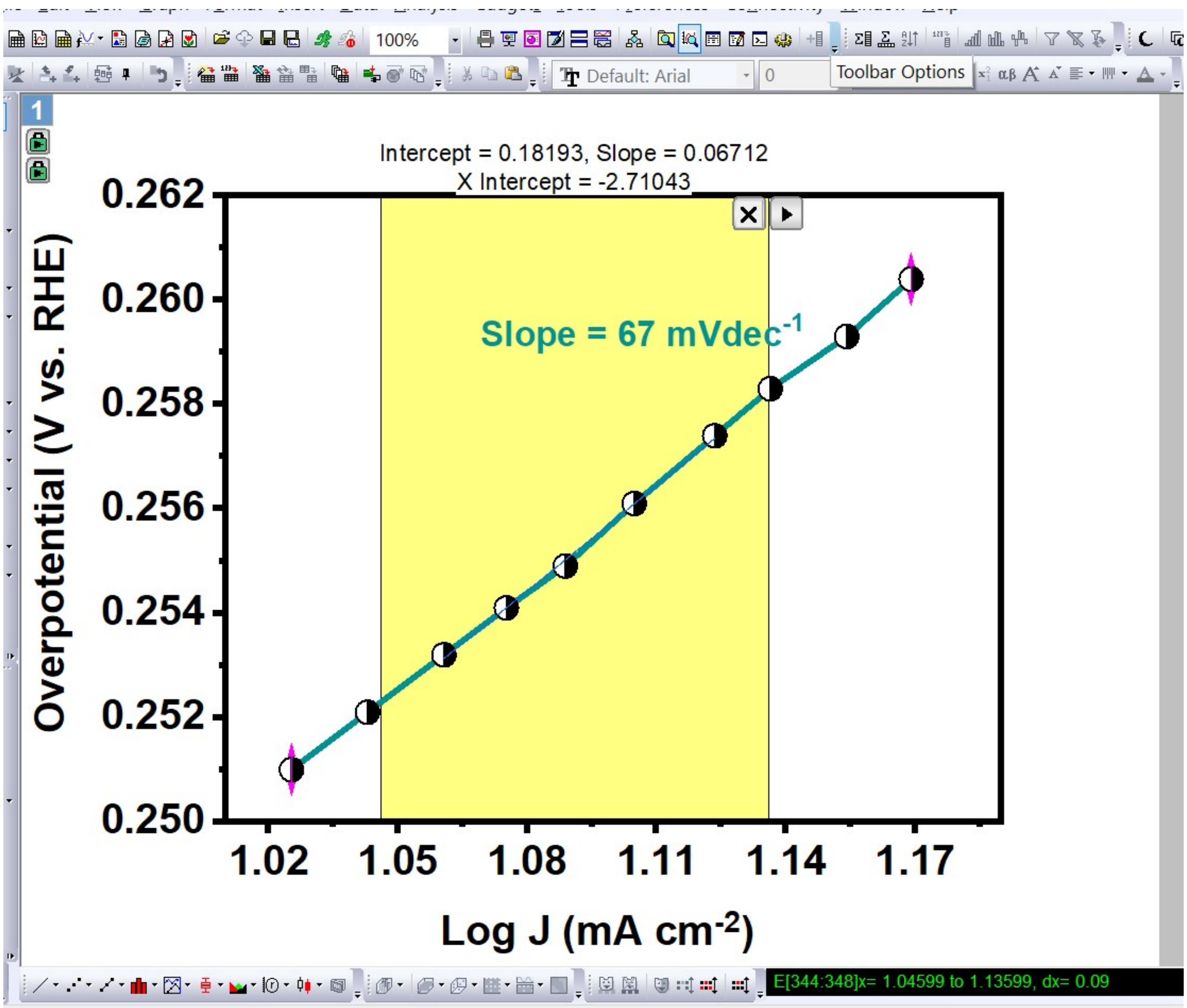

Figure S3. Linear fit graph for the Tafel Slope.

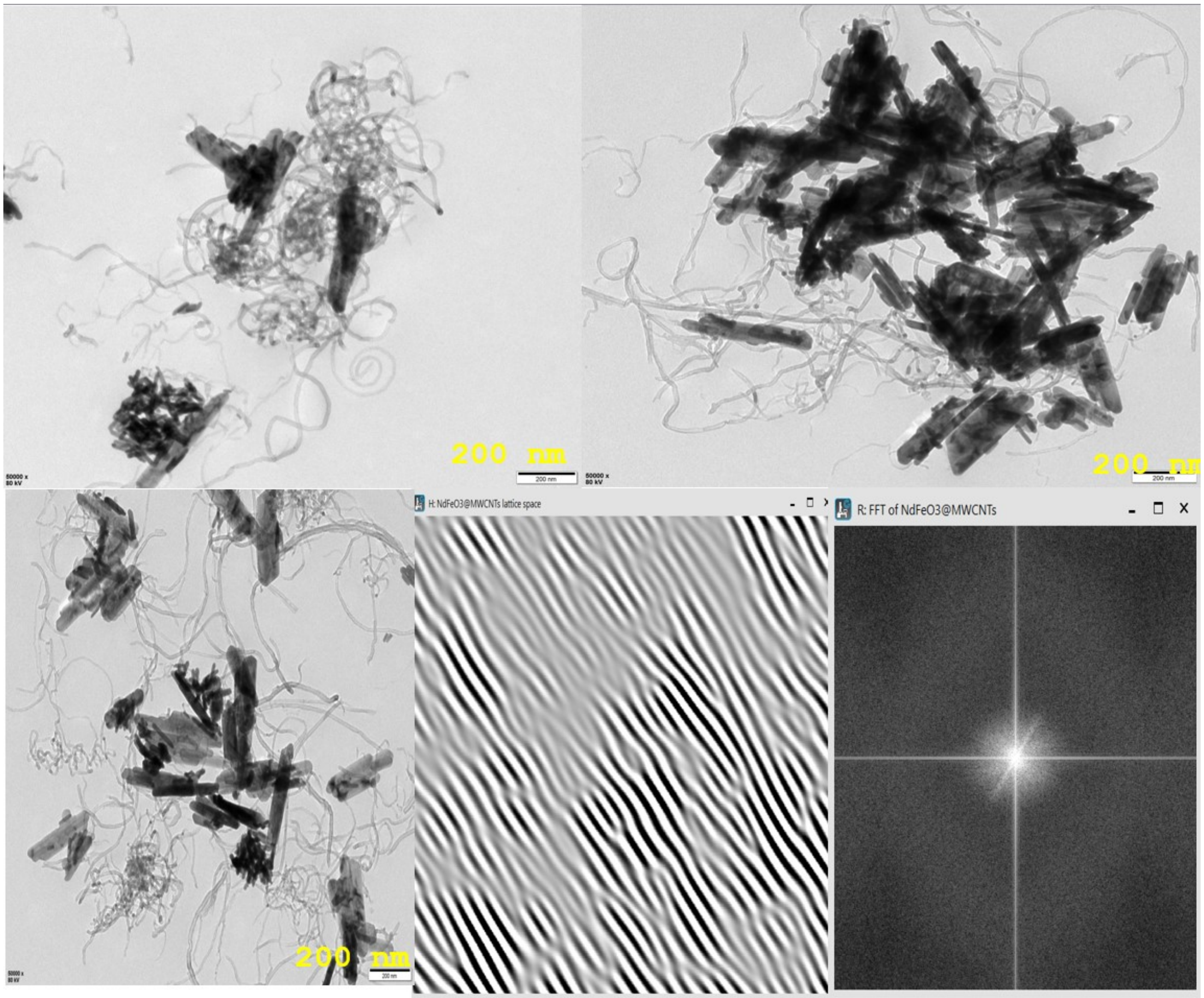

Figure S4. FFT and IFFT of CNTs and $NdFeO_3$ showing interlayers spacing in the middle bottom row.

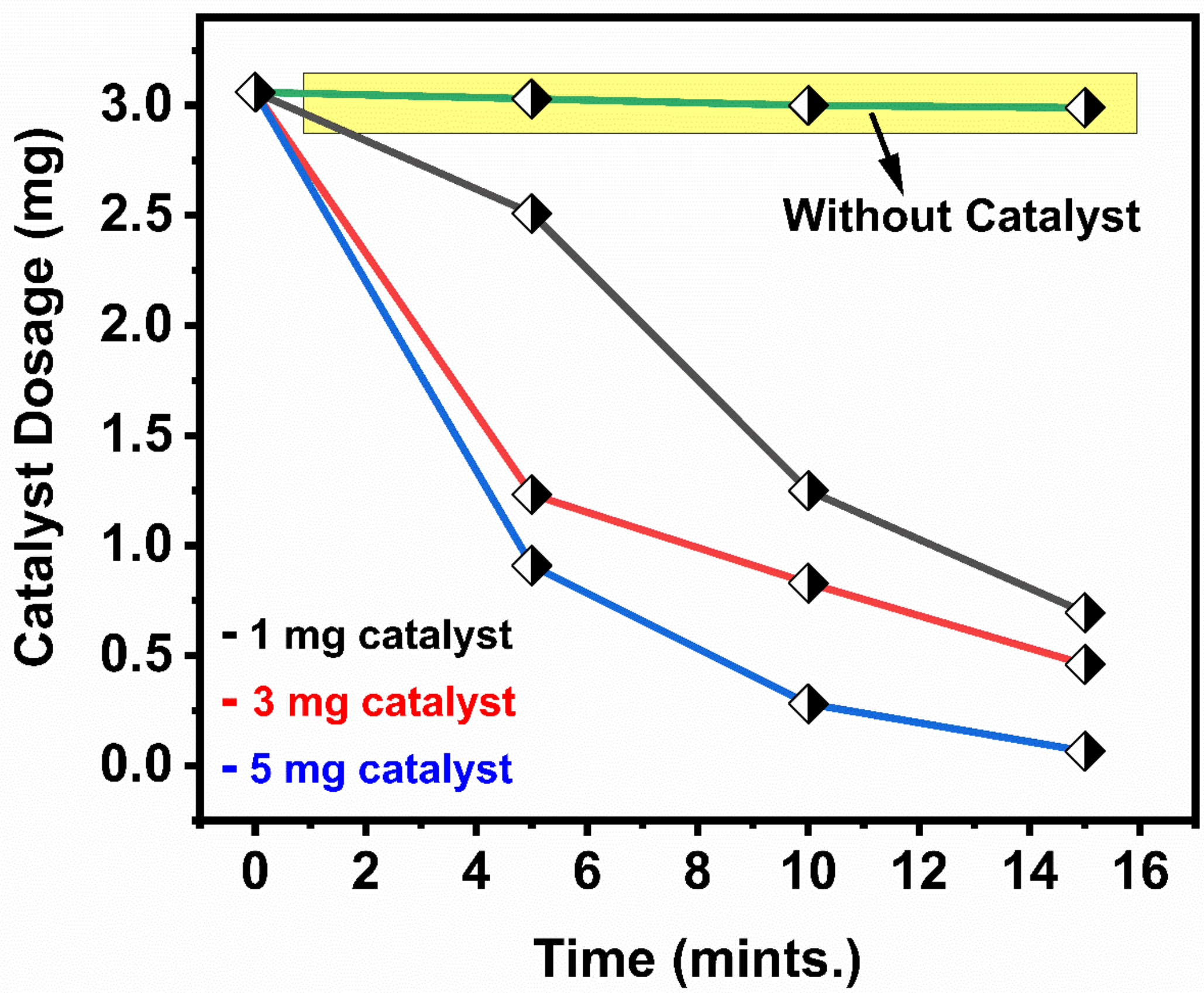

Figure S5. Effect of catalyst dosage on degradation efficiency

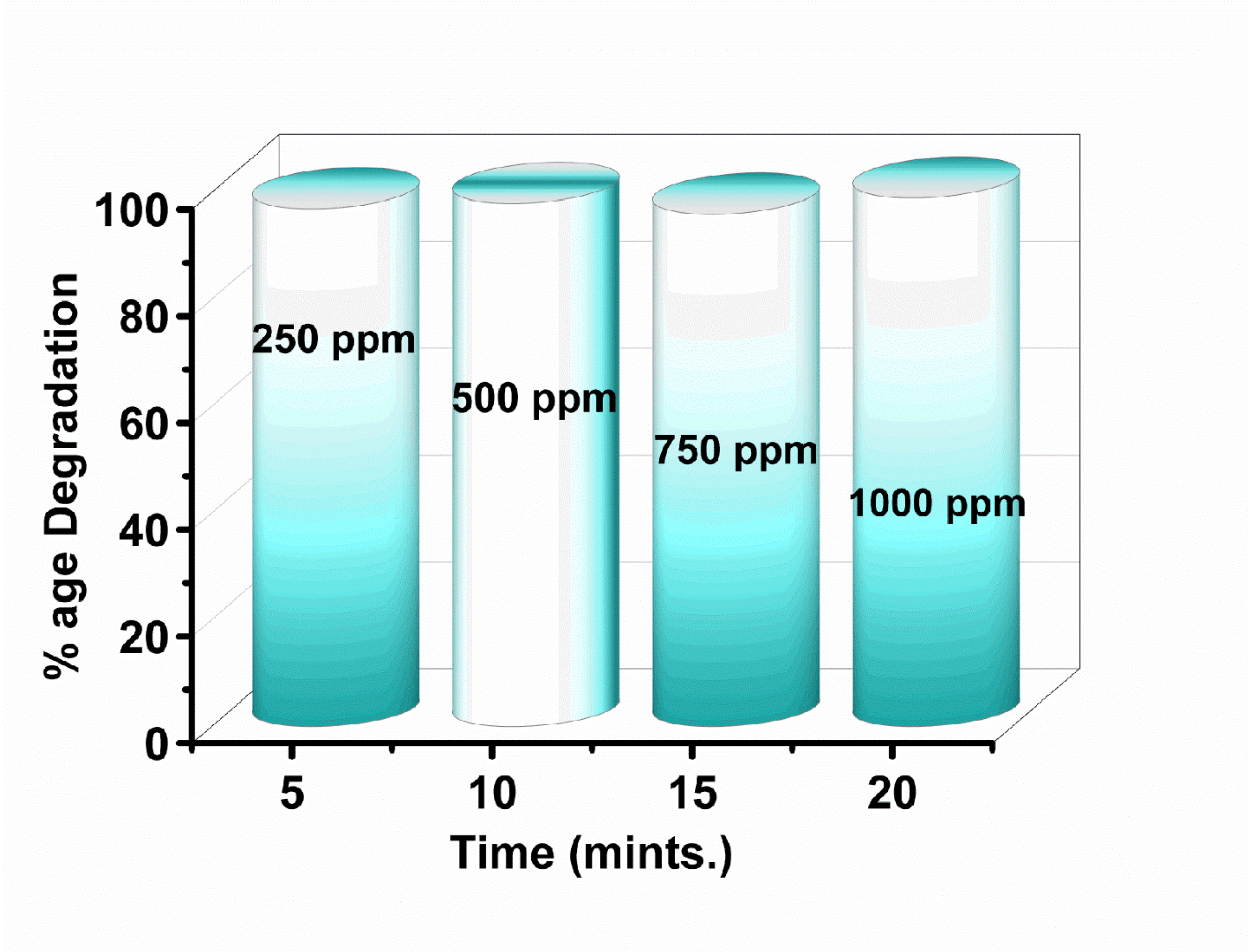

Figure S6. Effect of pollutant concentration

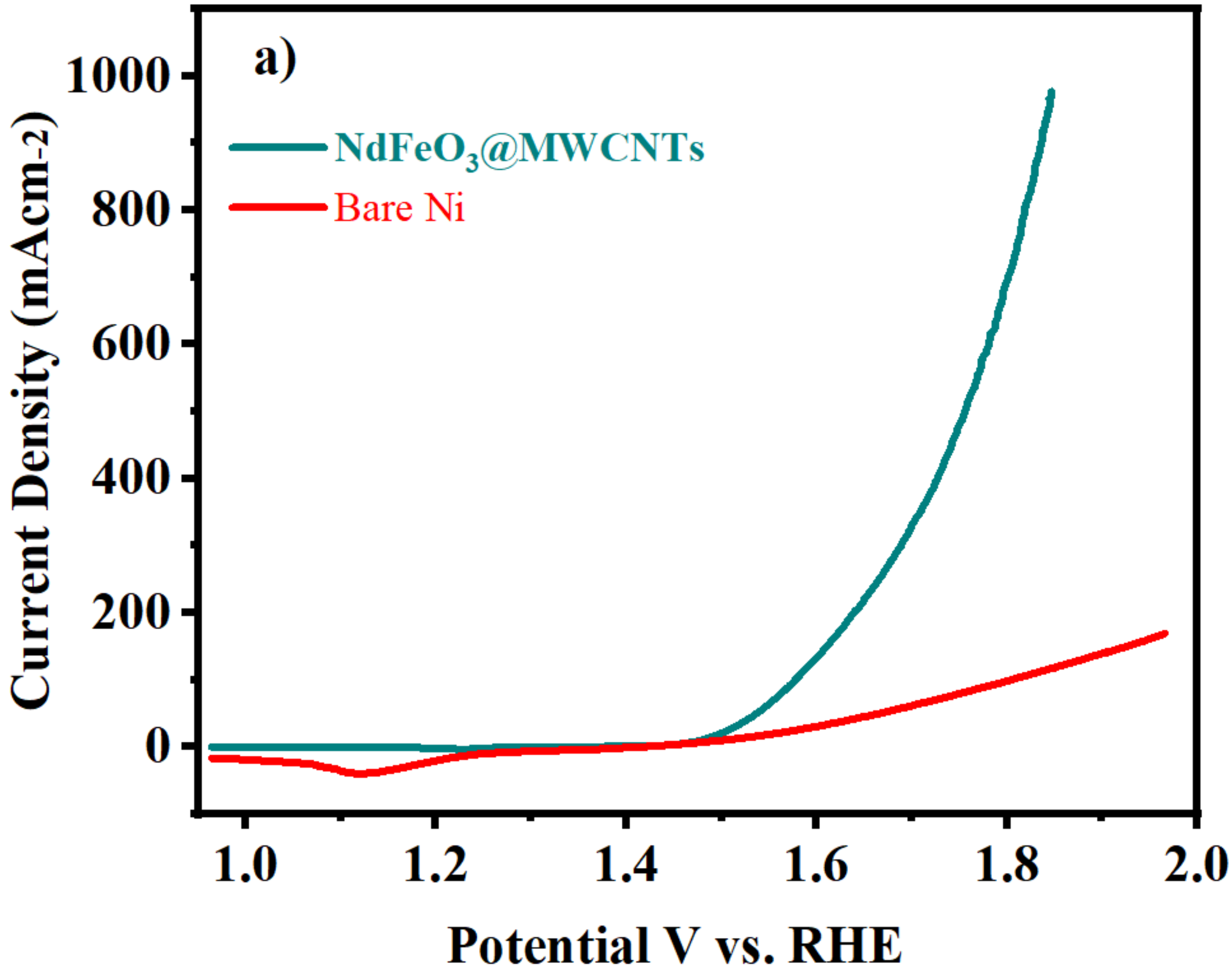

Figure S7: a) Shows LSV at a 5 mV/sec scan rate for composite and Bare Ni for comparison with and without catalyst

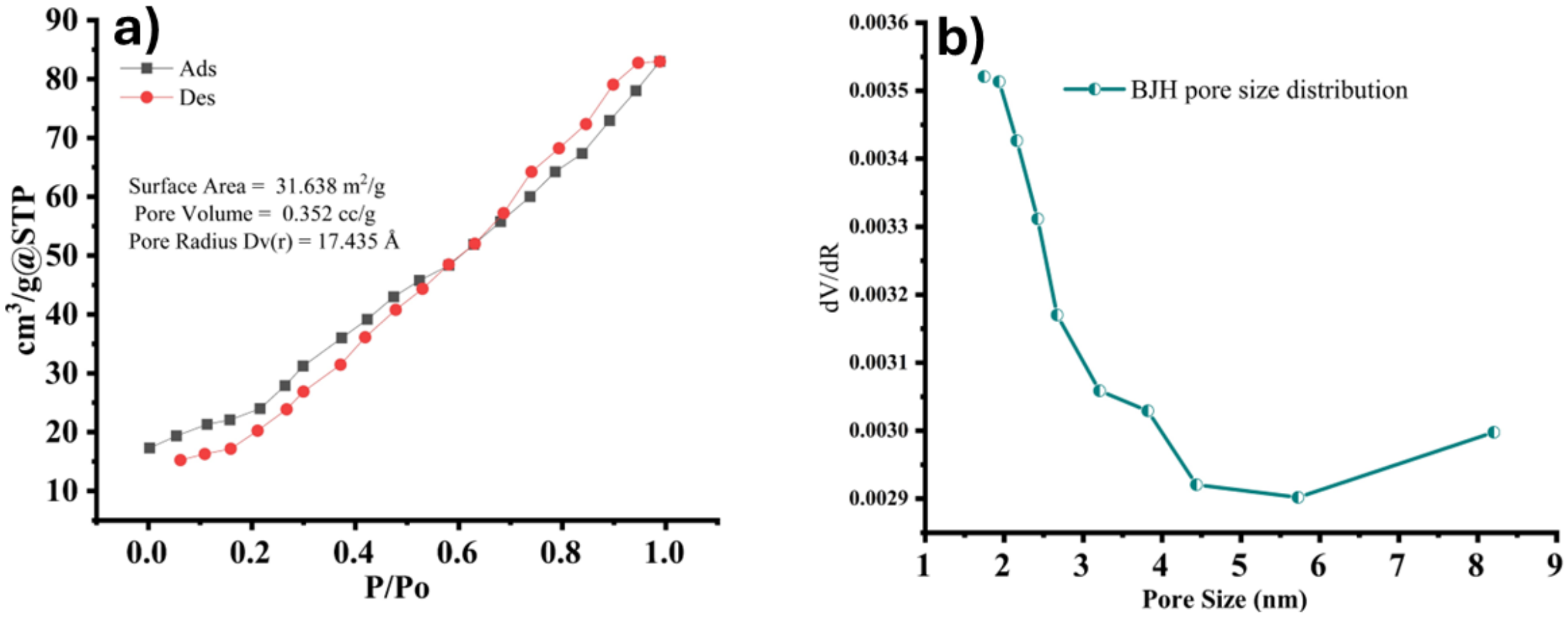

Figure S8. (a) Nitrogen adsorption-desorption isotherm and BET surface area analysis of $NdFeO_3$@MWCNTs nanocomposite. (b) BJH pore size distribution curve of $NdFeO_3$@MWCNTs nanocomposite.

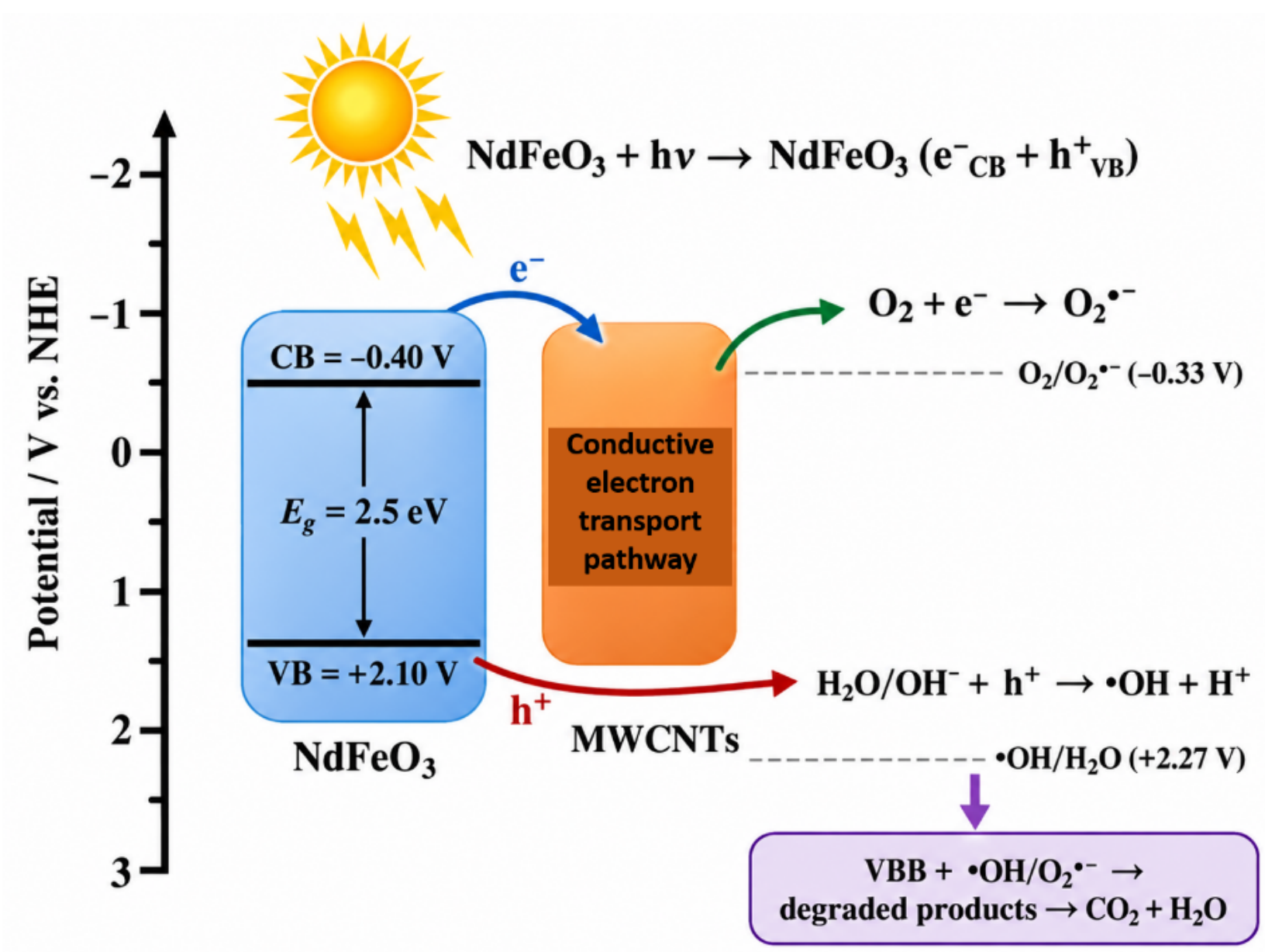

Figure S9: Proposed photocatalytic degradation mechanism of Victoria Blue B by NdFeO₃@MWCNTs nanocomposite showing band alignment, charge transfer pathway, reactive oxygen species generation, and dye degradation process.

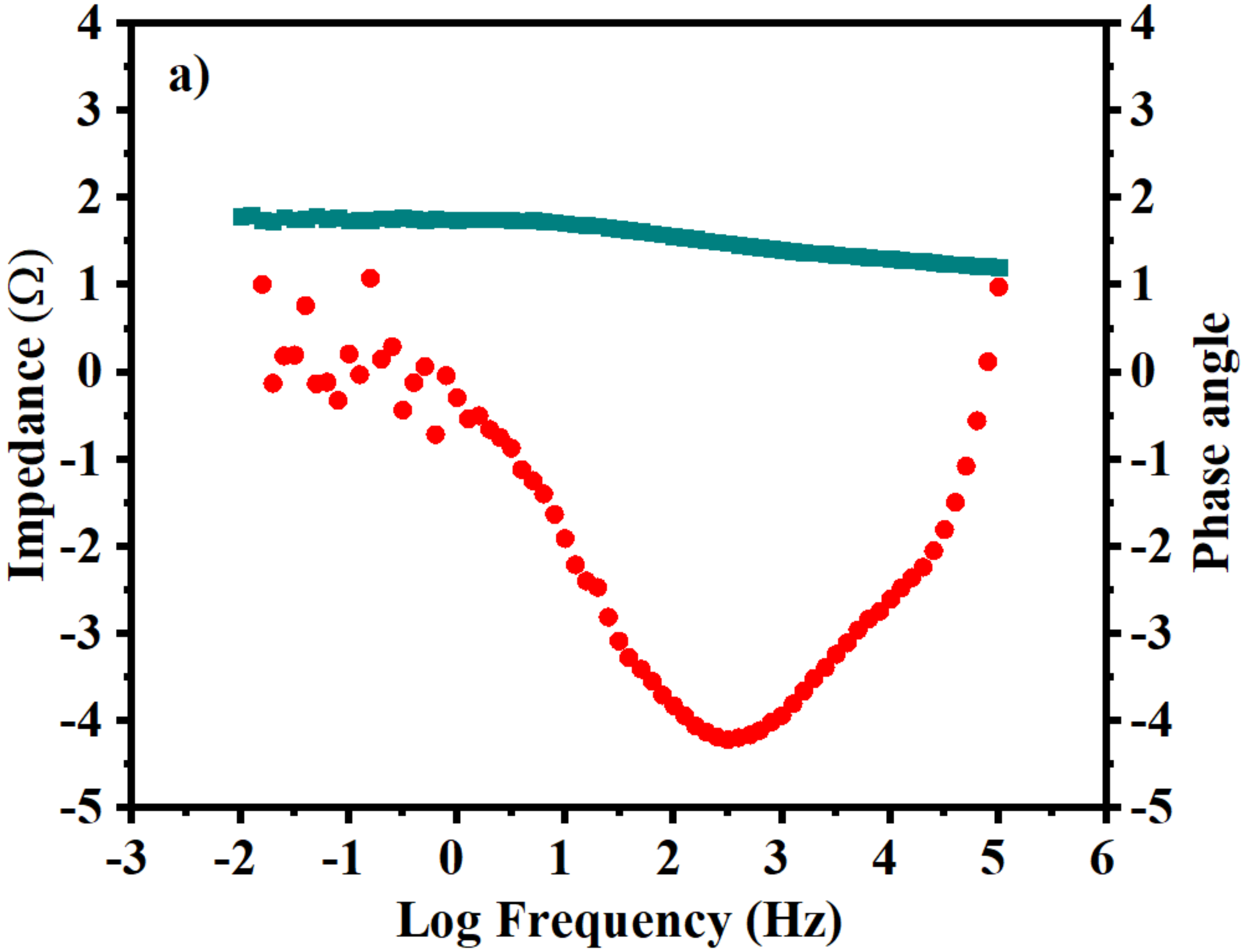

Figure S10 : a) Shows Bode plot representing impedance and phase angle against log of frequency

Table S2: Comparison of the photocatalytic performance of $NdFeO_3$@MWCNTs with previously reported perovskite-based photocatalysts

Photocatalyst	Target pollutant	Pollutant concentration	Light source	Degradation efficiency (%)	Time (min)	Reference
NdFe₂O₄@g-C₃N₄	Ciprofloxacin (CIP)	5 mg L⁻^{1}	150 W Xe lamp	100	NR*	[8]
Gd/Cu co-doped NdFeO₃	Crystal Violet	NR*	Photocatalytic irradiation	91.8	<50	[9]
NdFeO₃	Methylene Blue	15 mg L⁻^{1}	Visible light, 30 W LED	95	120	[10]
NdFe₀.₈₅Cu₀.₁₅O₃	Phenol	NR*	Visible light-assisted photo-Fenton	98.4	NR*	[11]
Cu-doped CaTiO₃	Methylene Blue	NR*	Visible light	99.4	120	[12]
$NdFeO_3$@MWCNTs	Victoria Blue B	250 mg L⁻^{1}	UV light	97	5	This work

Reference

1. Yang, J., T. Fujigaya, and N.J.S.R. Nakashima, *Decorating unoxidized-carbon nanotubes with homogeneous Ni-Co spinel nanocrystals show superior performance for oxygen evolution/reduction reactions.* 2017. **7**.
2. Roy, A., et al., *Influence of electrochemical active surface area on the oxygen evolution reaction and energy storage performance of MnO2-multiwalled carbon nanotube composite.* 2021. **45**: p. 16908 – 16921.

3. Thomas, A.A., A. Anupama, and M. Junaid Bushiri, *Solution combustion synthesis of NdFeO3 perovskite supported on graphitic carbon nitride as a bifunctional OER and HER electrocatalyst.* Materials Chemistry and Physics, 2026. **355**: p. 132214.
4. Omari, E. and M. Omari, *Enhancing catalytic activity of NdFeO3 perovskite by tuning A-site cation deficiency for oxygen evolution reaction.* International Journal of Hydrogen Energy, 2022. **47**(32): p. 14542–14551.
5. Sun, X., et al., *Sulfur Incorporated CoFe2O4/Multiwalled Carbon Nanotubes toward Enhanced Oxygen Evolution Reaction.* 2017. **247**: p. 843–850.
6. Wang, M., et al., *High quality bifunctional cathode for rechargeable zinc-air batteries using N-doped carbon nanotubes constrained CoFe alloy.* Journal of Colloid and Interface Science, 2024. **661**: p. 681–689.
7. Ullah, H., et al., *Bromine doped carbon encapsulated cobalt nanoparticles supported on carbon nanotubes for overall water splitting.* Journal of Power Sources, 2026. **673**: p. 239600.
8. Adewuyi, A. and R.A. Oderinde, *Synthesis of neodymium ferrite incorporated graphitic carbonitride (NdFe(2)O(4)@g-C(3)N(4)) and its application in the photodegradation of ciprofloxacin and ampicillin in a water system.* RSC Adv, 2023. **13**(8): p. 5405–5418.
9. Al-Abbad, E.A., et al., *Nanostructured codoped neodymium orthoferrite synthesized via the hydrothermal route for photocatalytic annihilation of crystal violet dye.* Ceramics International, 2024. **50**(24, Part C): p. 54943–54954.
10. Hosakote shankara, A., et al., *Facile synthesis of NdFeO3 perovskite for photocatalytic degradation of organic dye and antibiotic.* Materials Today: Proceedings, 2023. **75**: p. 15–23.
11. Phan, T. and T. Chu, *Enhanced Degradation of Phenol by Visible Light-Assisted Fenton Catalytic Activity of Copper-Substituted Neodymium Ferrite.* Environmental Processes, 2023. **10**.
12. Bhat, D.K., P.I. Uma, and U.S. Shenoy, *Exceptional light harvesting in copper doped CaTiO3 nanocuboids with surface nanosteps for the photo remediation of toxic Cr(VI) ions and dyes.* Journal of Alloys and Compounds Communications, 2024. **4**: p. 100030.
